# UHPLC-ESI-ORBITRAP-MS analysis of the native Mapuche medicinal plant palo negro (*Leptocarpha rivularis* DC. – Asteraceae) and evaluation of its antioxidant and cholinesterase inhibitory properties

**DOI:** 10.1080/14756366.2018.1466880

**Published:** 2018-05-07

**Authors:** Andrea Jiménez-González, Cristina Quispe, Jorge Bórquez, Beatriz Sepúlveda, Felipe Riveros, Carlos Areche, Edgar Nagles, Olimpo García-Beltrán, Mario J. Simirgiotis

**Affiliations:** a Facultad de Ciencias Naturales y Matemáticas, Universidad de Ibagué, Iquique, Colombia;; b Instituto de Etnofarmacología, Universidad Arturo Prat, Facultad de Ciencias de la Salud, Iquique, Chile;; c Laboratorio de Productos Naturales, Departamento de Química, Facultad de Ciencias Básicas, Universidad de Antofagasta, Antofagasta, Chile;; d Departamento de Ciencias Químicas, Universidad Andres Bello, Viña del Mar, Chile;; e Departamento de Química, Facultad de Ciencias, Universidad de Chile, Santiago, Chile;; f Instituto de Farmacia, Facultad de Ciencias, Universidad Austral de Chile, Valdivia, Chile;; g Center for Interdisciplinary Studies on the Nervous System, Universidad Austral de Chile, Valdivia, Chile

**Keywords:** Electrospray, antioxidants, metabolomics, Orbitrap, *Leptocarpha rivularis*

## Abstract

UHPLC/ESI/MS identification of organic compounds is the first step in the majority of screening techniques for the characterization of biologically active metabolites in natural sources. This paper describes a method for the fast identification and characterisation of secondary metabolites in *Leptocarpha rivularis* DC. (*Palo negro*) extracts by HPLC/UV (DAD)–Mass Spectrometry (HPLC/MS). The plant is used for the treatment of several diseases since pre-hispanic Mapuche times. Thirty-seven compounds were detected in the aqueous edible extract for the first time including 4 sesquiterpenes, 10 flavonoids, 9 oxylipins, 2 organic acids, and 11 phenolic acids. In addition, phenolic content antioxidant and cholinesterase inhibitory activities were measured for the first time using the edible infusion. The total polyphenol content of the infusion was 230.76 ± 2.5 mmol GAE/kg dry weight, while the antioxidant activity was 176.51 ± 28.84; 195.28 ± 4.83; and 223.92 ± 2.95 mmol TE/kg dry weight, for the DPPH, ABTS, and FRAP assays, respectively. The cholinesterase inhibitory activity was 7.38 ± 0.03 and 5.74 ± 0.06 mmol GALAE/kg, for the inhibition of acetylcholinesterase AChE and BChE, respectively, showing that this plant is a candidate for the isolation of compounds that can be useful for the treatment of neurodegenerative diseases. Furthermore, this plant could serve also as a raw material for the production of dietary supplements, due to its content of polyphenolic compounds.

## Introduction

**Figure 1.**
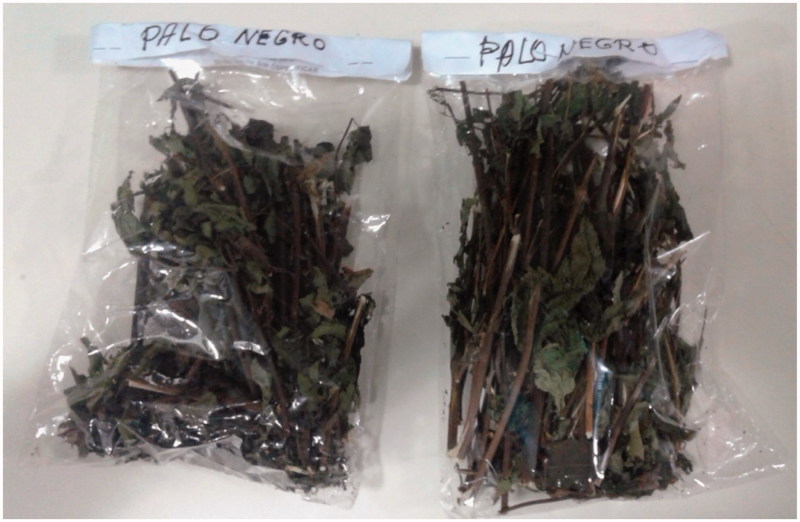
*L. rivularis* as it is sold in the street markets in Valdivia, Chile.

*Leptocarpha rivularis* DC. (Asteraceae), a native bush with the local name *Palo negro* in Chile, and *Cüdu-mamëll* in Mapudungun language: *kudü black*; *mamëll wood*, *tree*) is a medicinal South American native plant, which grows in the Valdivian forest, with exposure to large amounts of water and sun, generally growing in marshes or in permanent watercourses. This evergreen bush belongs to the family Asteraceae (Compositae) and to the genus *Leptocarpha*, which has only this species. This bush has been widely used in traditional Mapuche medicine for gastrointestinal and stomach ailments since pre-Hispanic times, and now it is largely sold in local markets and pharmacies for the prevention of cancer (Figure 1). Previous partial phytochemical studies of this plant showed the presence of sesquiterpenes^1^^,^^2^ and among them, the main active compound is the heliangolide leptocarpin^2^^,^^3^, which is an inhibitor of NF-kappa B and thus cytotoxic for cancer cells^3^. *Palo negro* is also a source of lipophilic compounds present in its essential oil, namely: alpha-thujone, beta-caryophyllene, and caryophyllene oxide, which were quantified mostly in the leaves^4^^,^^5^ and reported responsible for some of the antioxidant properties of the plant^6^. However, no chemical properties of the aqueous extract (the infusion, the edible medicinal form) was reported so far to the best of our knowledge.

In recent years, phenolic compounds gained great attention for the design of new nutraceuticals and phyto-pharmaceuticals, due to their important biological activities such as anti-inflammatory, antioxidant, anticancer, or antibacterial^7^^,^^8^. Indeed, the finding of herbs or medicinal plants with the presence of a high content of phenolics can be convenient to produce medicines to counteract oxidative stress conditions in the human body, and other diseases related to chronic inflammation.

On the other hand, diseases of the central nervous system (CNS) are responsible for 12% of worldwide deaths, and several people suffering from these diseases such as chronic neurodegenerative conditions (i.e. Alzheimer's disease (AD) and Parkinson's disease) are reported to have a poor quality of life^9^. So far, there are 30 million people affected with neurodegenerative conditions and these numbers will be rising by 2040^10^. China, Western Pacific countries, Eastern Europe, and the United States have the largest number of people affected by these diseases^11^. Consequently, in the last decade, there is a growing interest into new compounds’ discovery for the treatment or prevention of mentioned diseases, which could mimic neurotrophic factors, lower the oxidative stress status within the nervous system cells, increment neurotransmitters, or act as neuromodulators to accelerate neurogenesis^12^^,^^13^. Among the treatment strategies for those degenerative diseases are cholinesterase inhibitors such as galantamine^14^. Phenolic compounds present in extracts from a vast number of edible fruits, mushrooms, and herbs including *Salvia officinalis*, *Melissa officinalis*, *Laurus nobilis*, *Mentha suaveolens*, *Lavandula angustifolia*, *Lavandula pedunculata* showed also anticholinesterase (AChE) activity, and are considered rich sources of molecules useful for the prevention and treatment of those neurodegenerative diseases^15–17^.

In this work, we have performed antioxidant capacity and AChE inhibitory activity of some extracts from Palo Negro and in order to characterise chemically the extracts, we used modern state of the art analytical equipment, such as ultra HPLC hyphenated with diode array and high resolution quadrupole Orbitrap® mass spectrometry (UHPLC-DAD-Q-OT-MS).

Among the state of the art Orbitrap® instruments in the market, the Q-Exactive Focus can detect and quantify small organic compounds such as phenolics and others using a combination of diode array detection with an orbital trap (Orbitrap®), a quadrupole (Q) and a high-performance collision cell (HCD), allowing also high mass diagnostic MS fragments^18–22^. This tool was very important for our research in the last few years particularly, for the identification of phenolic metabolites in Chilean plants^23–26^. In the present article, the chemical fingerprinting of the medicinal plant’s infusion *L. rivularis* (Figure 1) was performed for the first time based on UHPLC-DAD coupled with high-resolution electrospray ionization Orbitrap® tandem mass spectrometry (UHPLC-DAD-Q-OT-MS). Since no previous studies were reported on antioxidant and cholinesterase inhibitory activity of *Palo negro* polar extracts and its edible infusions, those assays were performed to correlate the phenolic profiles with these important bioactivities.

## Materials and methods

### Plant material

*L. rivularis* DC. aerial parts were collected in Valdivia, in November 2016 and were identified by the botanist Alicia Marticorena from the University of Concepción, Chile. Voucher specimens are kept at the Natural Products Laboratory of the Universidad Austral de Chile, under reference number: LR20161115.

### Chemicals

UHPLC-MS Solvents, LC-MS formic acid and reagent chloroform were marketed from Merck (Santiago, Chile). Ultrapure water was obtained from a Millipore purification system (Milli-Q Merck Millipore, Santiago, Chile). UHPLC standards, (citric acid, caffeic acid, chlorogenic acid, gallic acid, isorhamnetin, diosmetin and rosmarinic acid, all standards with a purity higher than 95% for HPLC) were acquired either from Sigma Aldrich (Saint Louis, MO, USA), ChromaDex (Santa Ana, CA, USA), or Extrasynthèse (Genay, France). Folin-Ciocâlteu phenol reagent (2 N), reagent grade Na_2_CO_3_, AlCl_3_, HCl, FeCl_3_, NaNO_2_, NaOH, quercetin, trichloroacetic acid, sodium acetate, Tris-HCl buffer, Gallic acid, 2,4,6-Tris(2-pyridyl)-1,3,5-triazine (TPTZ), Trolox, nitroblue tetrazolium, xanthine oxidase, Acetylcholinesterase (AChE Electric eel, Type-VI-S, EC 3.1.1.7), butylcholinesterase (BChE from horse serum, EC 3.1.1.8) and DPPH (1,1-diphenyl-2-picrylhydrazyl radical) were acquired from Sigma-Aldrich Chemical Co. (Santiago, Chile).

### Sample preparation

Three grams (each extraction) of dried and milled aerial parts (leaves and stems) were separately macerated with ethanol (1 time, 250 ml at 25 °C), ethyl acetate (1 time, 250 ml, at 25 °C), for one hour. An infusion was prepared using 3 g of dried milled aerial parts adding deionized water (250 ml) at 85 °C, for 1 h. The solvents were concentrated *in vacuo* at 45 °C, and the infusion was lyophilized (Labconco 2.5 l) to obtain 229, 243, and 343 mg of ethanol, ethyl acetate, and aqueous extracts, respectively.

### UHPLC-DAD-MS instrument

The Dionex Thermo Scientific Ultimate 3000 UHPLC system (Bremen, Germany), hyphenated with a Thermo Q Exactive focus machine protocol was already reported^22^. For the analysis, each of the extracts were re-dissolved (2 mg per mL) in ethanol-distilled water (1:1 *v/v*) and 10 µL of filtered solution (PTFE filter) were injected in the instrument, with all specifications set as previously reported^22^.

### LC parameters

A binary gradient system with eluent (A) 0.1% formic acid in water, eluent (B) 0.1% formic acid in acetonitrile and the following gradient was used for the extracts: 10% B isocratic (0–1 min), 10–40% B (1–30 min), 40% B isocratic (30–33 min), 40–10% B (33–34 min), and 10% B isocratic (34–37 min). Liquid chromatography was performed using an UHPLC C18 column (Acclaim, 150 mm × 4.6 mm ID, 2.5 µm, Thermo Fisher Scientific, Bremen, Germany) operated at 25 °C. The detection wavelengths were 280, 254, 330, and 354 nm, (to detect mainly isoflavones-flavanones-flavanol, flavonols and phenolic acids) and PDA was recorded from 200 to 800 nm for peak characterization. Mobile phases were 1% aqueous formic acid solution (A) and acetonitrile acidified with 1% formic acid (B). The following gradient was used: 5% B isocratic (0–5 min), 5–30% B (5–10 min), 30% B isocratic (10–15 min), 30–70% B (15–20 min), 70% B isocratic (20–25 min), 70–5% B (25–35 min), and 15 min for column equilibration before injections. The flow rate was 1.00 ml min^−1^, and the volume injected was 10 ml. Standards and the resin extract dissolved in solvent were kept at 10 °C during storage in the auto-sampler.

### MS parameters

The HESI (heated electrospray ionization probe) parameters were as follows: sheath gas flow rate, 75 units; auxiliary gas unit flow rate, 20; capillary temperature, 400 °C; auxiliary gas heater temperature, 500 °C; spray voltage, 2500 V (for ESI-); and S lens, RF level 30. Full scan data in positive and negative were acquired at a resolving power of 70,000 FWHM (full width half maximum) at *m/z* 200. For the compounds of interest, a scan range of *m*/*z* 100–1000 was chosen; the automatic gain control (AGC) was set at 3 × 10^6^ and the injection time was set to 200 ms. The scan-rate was set at 2 scans s^−1^. External calibration was performed using a calibration solution in positive and negative modes. For confirmation purposes, a targeted MS-MS analysis was performed using the mass inclusion list, with a 30 s time window, with the Orbitrap spectrometer operating both in positive and negative modes at 17,500 FWHM (*m/z* 200). The AGC target was set to 2 × 10^5^, with the maximum injection time of 20 ms. The precursor ions were filtered by the quadrupole, which operated at an isolation window of *m*/*z* 2. The fore vacuum, high vacuum and ultrahigh vacuum were maintained at approximately 2 mbar, from 105 and below 1010 mbar, respectively. Collision energy (HCD cell) was operated at 30 kV. Detection was based on calculated mass and on retention time of target compounds, as shown in Table 1. The mass tolerance window was set to 5 ppm for the two modes. The HESI II and Orbitrap spectrometer parameters were set as stated previously^22^.

**Table 1.** Identification of metabolites by UHPLC-PDA-OT-MS in three extracts of leaves of *L. rivularis*.

Peak #	t_R_ (min.)	UV max	Tentative identification	Elemental composition [M–H]^−^	Theoretical mass (*m/z*)	Measured mass (*m/z*)	Accuracy (δppm)	MS^n^ ions (*m/z*)	Extract	References
1	1.47	210–272	Gluconic acid	C_6_H_11_O_7_^−^	195.05067	195.04993	3.82		A, B	^35^
2	3.87	–	Citric acid*	C_6_H_7_O_7_^−^	191.01959	191.01863	5.04		A, B	^36^
3	5.21	210–272	Chebulic acid	C_14_H_11_O_11_^−^	355.02959	355.03070	3.13	191.01933	A, B	^38^
4	7.55	172	Gallic acid*	C_7_H_6_O_5_^−^	169.01425	169.01639	3.81		A, B	^54^
5	9.79	207–309	Protocatechuic acid 4-*O*-glucoside	C_13_H_15_O_9_^−^	315.07106	315.07214	3.44		A, B	^40^
6	10.35	236–329	1,6 Dicaffeoyl-glucose	C_24_H_23_O_14_^−^	535.10913	535.10823	1.68		A, B	^41^
7	11.31	236–329	Chlorogenic acid (3-*O*-caffeoyl quinic acid)*	C_16_H_17_O_9_^−^	353.08781	353.08792	0.31	707.18102 [2M-H]^−^, 191.05579 (quinic acid)	A, B	^26^^,^^34^^,^^43^
8	11.45	236–329	Cryptochlorogenic acid, (4-*O*-caffeoyl quinic acid)	C_16_H_17_O_9_^−^	353.08781	353.08795	0.39	707.18103 [2M-H]^−^, 179.03465 (caffeic acid)	A, B	^43^
9	11.82	236–329	*Neo*-Chlorogenic acid, (5-*O*-caffeoyl quinic acid)	C_16_H_17_O_9_^−^	353.08781	353.08797	0.39	707.18105 [2M-H]^−^, 179.03465 (caffeic acid)	A, B	^43^
10	12.01	254–361	Kaempferol 3-*O*-glucose	C_21_H_19_O_11_^−^	447.09351	447.09329	2.94	153.01877	A, B	^46^
11	12.28	275–324	Isorhoifolin (apigenin 7-*O*-rutinose)	C_26_H_27_O_16_^−^	577.15594	577.15652	−0.99	255.02986 (apigenin)	A, B	^36^
12	12.45	255–355	Quercetin 3-*O*-glucose	C_21_H_19_O_12_^−^	463.08838	463.08838	2.75	301.03538 (quercetin)	A, B	^47^
13	12.86	255–355	Lonicerin (luteolin-7-*O*-neohesperidose)	C_26_H_27_O_16_^−^	593.15070	593.15010	1.01	285.04035 (luteolin)	A, B	^36^
14	13.28	236–329	3,4-Di-caffeoyl-quinic acid	C_25_H_23_O_12_^−^	515.11840	515.11932	1.78	353.08789 (caffeoyl quinic acid)	A, B	^43^
15	13.48	236–329	Isochlorogenic acid A; 3,5-Dicaffeoylquinic acid	C_25_H_23_O_12_^−^	515.11840	515.11957	2.26	353.08786 (caffeoyl quinic acid)	A, B, C	^43^
16	13.98	254–361	Luteolin-3-*O*-rhamnose	C_21_H_19_O_10_^−^	431.09727	431.09854	2.94	285.04083 (luteolin), 255.02951	A, B	^48^
17	14.34	236–329	Caffeic acid*	C_9_H_7_O_4_^−^	179.03458	179.03389	3.86	135.04445	A, B, C	^44^
18	14.87	255–355	3′,5′Di-*O*-methyl-myricetin	C_17_H_13_O_8_^−^	345.06171	345.06049	3.51	315.01486 (dehydrogenated myricetin)	A	^49^
19	15.50	215	9,10,12-Trihydroxy-octadecadienoic acid	C_18_H_31_O_5_^−^	327.21660	327.21790	3.95		A, B, C	^53^
20	16.98	255–355	3, 3´Di-*O*-methyl-myricetin	C_17_H_13_O_8_^−^	345.06171	345.06180	3.77	315.01486 (dehydrogenated myricetin)	A, B	^49^
21	18.57	210	9,10,12-Trihydroxyoctadecaenoic acid	C_18_H_31_O_5_^−^	329.23225	329.23358	4.04		A, B	^27^^,^^46^^,^^52^^,^^55^
22	19.25	235	11-Hydroxy-12-oxooctadeca-9,15-dienoic acid	C_18_H_29_O_4_^−^	309.20758	309.20604	4.99		A, B, C	^12^^,^^34^^,^^37^
23	19.43	235	11-Hydroxy-12-oxooctadeca-7, 9,15-trienoic acid	C_18_H_27_O_4_^−^	307.19183	307.19029	4.71		A, B, C	^12^^,^^34^^,^^37^
24	19.58	265–424	7-*O*-Methyl-8- prenyl-luteolin	C_21_H_19_O_6_^−^	367.11874	367.11761	3.07	285.04083 (luteolin),	A, B, C	^50^
25	19.85	225	Leptocarpin	C_15_H_19_O_3_^−^	361.16566	361.16577	0.30		A, B, C	^1–3^
26	19.98	266–419	8-Prenyl-kaempferol	C_20_H_17_O_5_^−^	337.10837	337.10842	0.14	217.05029, 134.03362	A, B	^50^
27	21.12	232	9-Hydroxy-octadecatrienoic acid	C_18_H_29_O_3_^−^	293.21112	293.21237	4.26		B	^12^^,^^34^^,^^37^
28	21.24	225	Leptocarpin dehydrated derivative	C_15_H_19_O_3_^−^	247.13287	247.13390	3.90		A, B, C	^1–3^
29	22.39	225	Leptocarpin dehydrated derivative	C_15_H_19_O_3_^−^	247.13397	247.13374	3.52		A, B, C	^1–3^
30	22.31	225	9-Hydroxy-octadecatetraenoic acid	C_18_H_27_O_3_^−^	291.19684	291.19814	−4.5		A, B, C	^12^^,^^34^^,^^37^
31	22.56	215	9-Hydroxy-octadecadienoic acid	C_18_H_31_O_3_^−^	295.22806	295.22677	4.35		A, B, C	^12^^,^^34^^,^^37^
32	22.78	225	8-Methoxy-13-hydroxy-9,11-octadecadienoic acid	C_19_H_33_O_4_^−^	325.23880	325.23734	3.15		A, B	^12^^,^^34^^,^^37^
33	23.36	246–310	Rosmarinic acid *	C_18_H_15_O_8_^−^	359.07769	359.07614	3.47		A, B, C	^45^
34	23.87	–	Ilicic acid	C_15_H_23_O_3_^−^	251.16527	251.16518	4.00		A, B, C	^37^
35	24.7		Diosmetin (4′-*O*-methyl-luteolin)*	C_16_H_11_O_6_^−^	299.05618	299.05501	3.90	285.04083 (luteolin)	A, B, C	^51^
36	26.32	212	Dihydroxyoctadecadienoic acid	C_18_H_31_O_4_^−^	311.22302	311.22169	4.29		A, B, C	^12^^,^^34^^,^^37^
37	27.27	254–354	Isorhamnetin*	C_15_H_7_O_8_^−^	315.01398	315.01354	1.37		A, B, C	^26^

*Identified by spiking experiments with an authentic compound. MS^n^: Daughter ions.

A: Ethanolic extract; B: aqueous extract; C: ethyl acetate extract.

### Total phenolic and flavonoids content

The analysis of total phenolic content (TPC) was based on the study performed by Simirgiotis et al.^27^ with slight modifications. To 100 µL of extract, 940 µL of Milli-Q water and 480 µL of the reagent Folin-Ciocâlteu (10%, Merck, Santiago, Chile) were added to a test tube, mixed using a vortex. The prepared tube was allowed to react for 5 min, then 480 µL of 10% sodium carbonate was added. The mixture was incubated at room temperature for 30 min in the absence of light. Absorbance was then measured at 765 nm using a UV-Vis. spectrophotometer (Spectroquant Pharo 300 Merck, Santiago, Chile), using a Milli-Q water blank with all other reagents without the sample. The absorbance values were replaced in the equation of a standard curve using gallic acid (µmol × L^−1^). The total phenolic content was then expressed as millimoles of gallic acid equivalents per kilogram of dry sample weight (mmol GAE/kg extract). The aluminum chloride method was used for the determination of the total flavonoid content^27^. For this test, 1 ml of the filtered infusion was mixed into a 10 ml volumetric bottle with 4 ml of distilled water. Then, 0.3 ml of 5% NaNO_2_ was added to the bottle and hand shook for 10 s. After 5 min, 0.3 ml 10% AlCl_3_ was added to the sample mixture. At the sixth minute, 2 ml of the 1 M NaOH solution was added, and the volume filled to 10 ml with water. The absorbance was then measured at 510 nm, using UV-Visible spectrophotometer, after diluting the sample ten times (1:10 *v/v* sample mixture:water). Flavonoid content (TFC) was calculated using a quercetin standard calibration curve (concentrations ranging from 16.0 to 800.0 µg/mL, *R*^2^ = 0.995). Results were expressed as micromoles quercetin equivalents per kilogram of dry weight (µmol QE/kg dry weight).

### Antioxidant assays

#### DPPH cation radical test

The capturing capacity of the DPPH^•^ radical was evaluated by the decolorization method developed by Brand-Williams et al.^28^, and modified by Kim et al.^29^. Briefly, to 400 µl of extract, (at 2 mg/mL), 2 ml of a 100 µM methanolic DPPH solution (Absorbance: 1.10 ± 0.02, at 517 nm) were added. The mixture was homogenised using a vortex, and kept at room temperature for 20 min in the absence of light. The percentage of discoloration of the DPPH moiety was calculated by reading the absorbance at 517 nm, and the values obtained converted to percent inhibition of the DPPH moiety using the following:

$$Percentage\;Inhibition=\left[1-\frac{S.A.}{B.A.}\right]\times 100$$

where S.A. is sample absorbance and B.A. is blank absorbance.

The values were replaced in the Trolox standard curve equation (µmol × L^−1^). The results were expressed as Trolox equivalent antioxidant capacity (TEAC), in millimoles Trolox Equivalents per kilogram of dry weight (mmol TE/kg dry weight)

#### Bleaching test with the cationic radical ABTS^• +^

The capturing capacity of the ABTS^•+^ radical was evaluated by the decolorisation method developed by Re et al. 1999 and modified by Kuskoski et al. 2004^30^. The radical ABTS^•+^ is generated chemically by the oxidation of ABTS with potassium persulfate after 16 h of incubation at room temperature in the dark. Briefly, to 2 ml of the ABTS^•+^ solution (previously adjusted with 80% methanol in ultrapure water, in order to obtain an absorbance of 0.70 ± 0.02 at 734 nm), 200 µL of the extract was added (prepared at 2 mg/mL), mixed using a vortex and allowed to react in the dark at room temperature. The absorbance was then measured at 765 nm after 6 min of incubation, and the values obtained converted to % inhibition of the ABTS^•+^ radical and substituted in the Trolox standard curve equation (µmol × L^−1^). The results were expressed as millimoles of Trolox equivalents per kilogram of dry weight (mmol TE/kg dry weight).

#### Ferric reduction antioxidant power test (FRAP)

For the FRAP test, the methodology proposed by Benzie and Strain^31^, was used with some slight modifications. Briefly, to 200 µL of extract (2 mg/mL), 2 ml of the FRAP solution was added and mixed using a vortex, allowing to react in the dark at room temperature for 5 min. The absorbance measurement of the colored Fe-TPTZ complex was performed at 595 nm. Absorbance values were replaced in the Trolox standard curve equation (µmol × L^−1^). The results were expressed as Trolox equivalents (TE), in millimoles Trolox per kilogram of dry weight (mmol TE/kg dry weight).

#### Superoxide anion scavenging assay

The xanthine oxidase enzyme yields superoxide anion radical (O_2_**^−^**) *in vivo* by oxidation of reduced products from intracellular ATP metabolism. Xanthine oxidase has been reported to increase its activity during oxidative stress and produce uric acid and superoxide anion radical. This radical reduces the blue nitro tetrazolium dye (NBT), to mono- and di-formazan generated in the biological system consisting of the enzyme xanthine oxidase (XOD) and hypoxanthine at pH 7.4 forming a blue chromophore, which absorbs at 520 nm. Superoxide anion scavengers reduce the speed of generation of the chromophore. The assay involved the addition of 20 µl of sample solution (extract at 100 mg/mL) and 160 µl of a reaction mixture containing 50 mM potassium phosphate buffer (pH 7.4), 0.2 mM NBT, 0.6 mM hypoxanthine, and 1 mM EDTA. The reaction started by the addition of 20 µl of XOD (200 mU/mL) and incubation for 8 min at 37 °C. Allopurinol was used as a positive control. Extracts dissolved in water (containing 5% DMSO for solubility) were used as sample solutions. The Superoxide anion trapping activities (SAA) of the extracts were measured spectro-photometrically as previously described^32^.

#### Cholinesterase (ChE) inhibition

Cholinesterase inhibitory activity was performed using Ellman’s method, as stated previously^33^^,^^34^. The enzyme cholinesterase hydrolyses the substrate acetylthiocholine and results in the product thiocholine, which reacts with Ellman’s reagent (DTNB) to produce 2-nitrobenzoate-5-mecraptothiocholine and 5-thio-2-nitrobenzoate, which can be detected at 405 nm. Briefly, extracts obtained were evaporated under reduced pressure and further kept in a vacuum desiccator to fully remove traces of solvents. DTNB was dissolved in the buffer Tris-HCl buffer at pH 8.0 containing 0.1 M NaCl and 0.02 M MgCl_2_. Then, a filtered (PTFE) sample solution in deionised water (50 µL, 2 mg/mL) was mixed with 125 µL of 5-dithio-bis(2-nitrobenzoic) acid (DTNB), acetylcholinesterase (AChE), or butyrylcholinesterase (BChE) solution (25 µL) dissolved in Tris-HCl buffer at pH 8.0placed in a 96-well microplate and incubated for 15 min at 25 °C. The reaction was initiated with the addition of acetyl-thiocholine iodide (ATCI) or butyryl-thiocholine chloride (BTCl) (25 µL). In addition, a blank was prepared by adding the solution sample to all reagents without the enzyme(s) (AChE or BChE) solutions. The sample and blank absorbance were then recorded at 405 nm after 10 min of incubation at 25 °C. The absorbance of the blank was subtracted from the absorbance of the sample and the cholinesterase inhibitory capacity was expressed as galantamine equivalents (calibration range (5–70 µg/ml) per g extract.

#### Statistical analysis

The statistical analysis (ANOVA, five times for each determination) set using the originPro 9.0 software package (Originlab Corporation, Northampton, MA, USA).

### High resolution mass-mass metabolite identification in L. Rivularis

Electrospray Orbitrap became a versatile and very rapid tool for the characterisation of phenolics in medicinal plants. This state of the art technique was used to determine the metabolomic profiles of *L. rivularis* and to set up chemical fingerprints that could be useful for chemotaxonomy and identification of the plant material, since other species are known by that local name in Latin countries.

**Figure 2.**
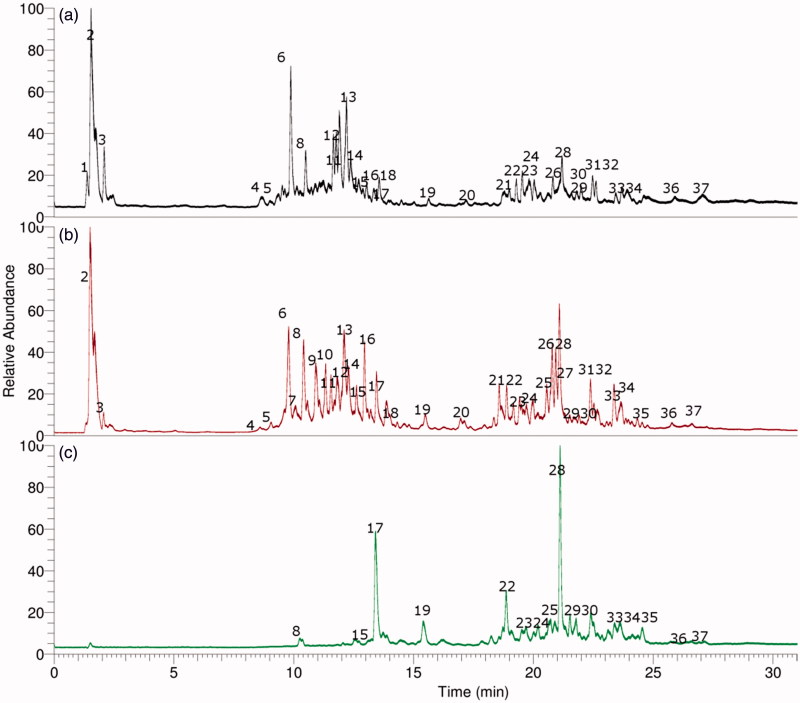
UHPLC Chromatograms of *L. rivularis* extracts, (a) ethanolic extract, (b) aqueous infusion (c) ethyl acetate extract.

Figure 2 shows the total ion current chromatograms (TIC) of *L. rivularis* extracts as: (a) ethanolic extract, (b) aqueous infusion, (c) ethyl acetate extract and Figure S1 shows the full HR-MS spectra and structures of some representative compounds. Conversely and as expected, the ethyl acetate extract showed a poorer chemical profile for these bioactive compounds. We have explained below the rapid metabolome analysis of these three extracts (ethanolic extract, ethyl acetate extract and infusion) prepared from this species.

#### Aliphatic organic acids

Two compounds (Figure 2) were identified as simple aliphatic organic acids, the first one was peak 1 showing a pseudomolecular ion at *m/z* 195.04993, which was identified as gluconic acid (C_6_H_11_O_7_^−^)^35^, and the second one was citric acid (C_6_H_7_O_7_^−^, peak 2)^36^.

#### Sesquiterpenes

Peak 25 with a [M–H]^−^ ion at *m/z* 361.16577 was characterised as leptocarpin (C_15_H_19_O_3_^−^)^3^ and peaks 28 and 29, with [M–H]^−^ ions at *m/z* 247.13390 and 247.13374 matching the formula: C_15_H_19_O_3_^−^ were assigned to dehydrated derivatives of leptocarpin (Figure S1g). Furthermore, peak 28 was one of the main compounds (Figure 2) in the aqueous extract that could be responsible at least in part, of the anticancer activity reported for this plant^1^^,^^3^. Peak 34 with a [M–H]^−^ ion at *m/z* 251.16518 (Figure S1k), was identified as ilicic acid (C_15_H_23_O_3_^−^)^37^.

#### Phenolic acids

Peak 3 with a [M–H]^−^ ion at *m/z* 355.03070 was identified as chebulic acid (C_14_H_11_O_11_^−^)^38^. Peak 4 showing a [M–H]^−^ ion at *m/z* 169.01639 was identified as gallic acid (C_7_H_5_O_5_^−^)^39^, while peak 5 was identified as protocatechuic acid 4-*O*-glucoside (C_13_H_15_O_9_^−^)^39^^,^^40^, and peak 6, which showed a [M–H]^−^ ion at *m/z* 535.10823 was identified as 1,6-dicaffeoyl-glucose (C_24_H_23_O_14_^−^)^41^. Furthermore, peaks 7–9 were identified as isomers of chlorogenic acid (3, 4, and 5 *O*-caffeoyl-quinic acids, respectively) (C_16_H_17_O_9_)^42^^,^^43^, all showing diagnostic [2 M–H]^−^ ions at around *m/z* 707 and daughter ions at *m/z* 179.03465 (caffeic acid)^26^^,^^39^. Peaks 14 and 15, showing pseudomolecular ions at *m/z* 515.11932 and 515.11957, respectively and yielding MS^2^ fragments at *m/z* 353 (caffeoyl quinic acid) were identified as di-caffeoyl quinic acid isomers^44^ (C_25_H_23_O_12_^−^, Figure S1c). Finally, peak 17 was characterised as caffeic acid (C_9_H_7_O_4_^−^) and peak 33 with a [M–H]^−^ ion at *m/z* 359.07614 (Figure S1j) was identified as the anti-inflammatory compound rosmarinic acid (C_18_H_15_O_8_^−^)^45^.

#### Flavonoids

Some compounds were tentatively characterised as flavonoid glycosides, while some were identified as diosmetin and luteolin derivatives. The UV spectra of quercetin, kaempferol, luteolin, and diosmetin solutions in methanol are typical for flavones, and the absorption maxima are observed in the range from 240 to 400 nm. The UV maxima are attributed to bands related to absorption involving an A-ring benzoyl system (usually 240–280 nm) and a B-ring cinnamoyl system (300–380 nm). Accordingly, peak 10 with a ion [M–H]^−^ at *m/z* 447.09329 was identified as kaempferol 3-*O*-glucose (C_21_H_19_O_11_^−^)^46^, peak 11 with a anion [M–H]^−^ at *m/z* 577.15652 () and an apigenin MS^2^ ion at *m/z* 255.02986 was labeled as isorhoifolin (apigenin 7-*O*-rutinose, C_26_H_27_O_16_^−^, Figure S1a)^36^, peak 12 with a [M–H]^−^ ion at *m/z* 463.08838 was identified as quercetin 3-*O*-glucose (C_21_H_19_O_12_^−^)^47^ and peak 13 with a anion [M–H]^−^ at *m/z* 593.15010 (Figure S1b) as lonicerin (luteolin-7-*O*-neohesperidose, C_26_H_27_O_16_^−^)^36^ and peak 16 as luteolin rhamnose (Figure S1d)^48^. Peaks 18 and 20 were identified as the isomers: 3′,5′di-*O*-methylmyricetin^49^ and 7,3′di-*O*-methylmyricetin (C_17_H_13_O_8_^−^), respectively. Peak 26 with UV max characteristic of kaempferol (λ max 266–419 nm) and with a ion [M–H]^−^ at *m/z* 337.10837 (Figure S1f) was identified as 8-prenyl-kaempferol (C_20_H_17_O_5_^−^) and peak 24 (Figure S1e) was tentatively identified as the related compound 7-*O*-methyl-8-prenyl luteolin (C_21_H_19_O_6_^−^), both prenylated flavones^50^. Finally, peak 35 was identified as diosmetin (C_16_H_11_O_6_^−^, Figure S1l)^51^ and peak 37 as isorhamnetin (C_15_H_7_O_8_^−^)^36^.

#### Oxylipins

Several compounds were identified as the dietary polyhydroxylated unsaturated fatty acids known as oxylipins^27^^,^^52^, important antioxidant fatty acids component of foods. Peak 19 and 21 with ions [M–H]^−^ at *m/z* 327.21790 and 329.23358 were identified as 9,10,12-trihydroxy-octadecadienoic acid and 9,10,12-trihydroxyoctadecaenoic acid^53^ respectively (C_18_H_31_O_5_^−^ and C_18_H_31_O_5_^−^). In the same manner, peaks 22 and 23 (HESI-MS: 309.20604 and 307.19029 Daltons) were determined as 11-hydroxy-12-oxooctadeca-9,15-dienoic acid and 11-hydroxy-12-oxooctadeca-7,9,15-trienoic acid, respectively. Peak 27 was assigned to 9-hydroxy-octadecatrienoic acid (C_18_H_29_O_3_^−^) while peaks 30 and 31 as 9-hydroxy-octadecatetraenoic acid (295.22677, C_18_H_27_O_3_^−^Figure S1h) and 9-hydroxy-octadecadienoic acid (C_18_H_31_O_3_^−^), respectively. Finally, peak 32 with a ion [M–H]^−^ at *m/z* 325.23734 (Figure S1i) was identified as 8-methoxy-13-hydroxy-9,11-octadecadienoic acid (C_19_H_33_O_4_^−^) and peak 36 with a ion [M–H]^−^ at *m/z* 311.22169 as dihydroxyoctadecadienoic acid (C_18_H_31_O_4_^−^)^27^^,^^52^.

#### Antioxidant capacities

In this study, several assays (DPPH, ABTS, FRAP, and SA) were performed in order to quantify the antioxidant capacity of some *Palo negro* extracts (Table 2). The antioxidant capacity measured by DPPH method was 176.51 ± 28.84 mmol TE/kg and in the FRAP assay, the infusion exert a value of 223.92 ± 2.95 mmol TE/kg. Eleven antioxidants were identified as phenolic acids (peaks 3–9, 14, 15, 17, and 33) and 10 were flavonoids (peaks 10–13, 18, 20, 26, 24, 35, and 37) that could be responsible for the antioxidant capacity presented. Furthermore, the nine oxylipins detected (peaks 22, 23, 27, 30, 31, 32, and 36), as well as the two organic acids (peaks 1 and 2) could contribute to the antioxidant activity. In addition, the total phenolic content measured spectroscopically (320.49 ± 3.58 mmol GAE/kg) of the infusion (medicinal form used traditionally) was higher to that already reported for the hydroalcoholic extract (water:ethanol 80:20 *v/v*) of this plant (3.7 ± 0.02 mg GAE/g, or 21.76 mmol GAE/kg)^4^. The difference could be explained considering the extraction method used or stage of the plant collected. The TPC and TFC values were also higher than those reported for the bioactive-containing compounds plants *Lathyrus cicera* and *Lathyrus digitatus*^56^.

**Table 2.** Content of phenolics and antioxidant capacities of *L. rivularis* extracts evidenced as the bleaching of the DPPH and ABTS radicals, FRAP (ferric reducing antioxidant power), SA (superoxide anion scavenging capacity), Total Phenolic Content (TPC), Total Flavonoids Content (TFC) and inhibitory activity of ACHe: acetylcholinesterase and BCHe: butylcholinesterase enzymes.

Sample	DPPH^a^	ABTS^a^	FRAP^b^	TPC^c^	TFC^d^	SA^e^	ACHe^f^	BCHe^f^
Ethanol	168.39 ± 23.87^a^	186.72 ± 7.09^b^	201.37 ± 4.91	267.43 ± 8.38	170.78 ± 6.92	62.59 ± 4.42^c^	1.45 ± 0.06^c^	1.28 ± 0.08
Aqueous	176.51 ± 28.84^a^	195.28 ± 4.83^b^	223.92 ± 2.95	320.49 ± 3.58	230.76 ± 2.5	75.18 ± 9.63^c^	2.12 ± 0.03^c^	1.65 ± 0.06
EtOAc	120.86 ± 23.89	132.49 ± 6.12	141.86 ± 6.64	140.98 ± 2.87	150.49 ± 0.46	48.73 ± 7.38	0.89 ± 0.03	0.72 ± 0.04

^a^Antiradical activities are expressed as mmol Trolox/kg dry weight.

^b^Ferric reducing power expressed as mmol Trolox/kg dry weight.

^c^Total phenolic content (TPC) expressed as mmol GAE/kg dry weight.

^d^Total flavonoid content (TFC) expressed as mmol QE/kg dry weight.

^e^SA is expressed as percent inhibition at 100 μg/mL.

Values in the same column marked with the same letter are not significantly different (at *p* < .05).

^f^Expressed as mg GALAE: galantamine equivalents per g extract.

#### Enzyme inhibitory properties

Acetylcholinesterase enzyme (AChE) plays a major role in the activity of the central and peripheral nervous systems, because it catalyses the hydrolysis and inactivation of the acetylcholine neurotransmitter, yielding choline and acetate. Cholinesterase inhibitors improve the cholinergic function of Alzheimer’s disease (AD), preserving the levels of acetylcholine, and, therefore have become the standard approach in the symptomatic treatment of AD. Those compounds, such as donepezil, galantamine, and rivastigmine, delay the degradation of the acetylcholine released into the synaptic clefts and, thus reinforce cholinergic neurotransmission. In this context, enzyme inhibitory assays have become, in the recent years, very useful tools to assess the potential health benefits of herbal medicines, fruits, and related biological materials, for the development of functional foods or dietary supplements^56^^,^^57^. Moreover, most usual assays involve key enzymes relevant in chronic neurodegenerative conditions such as AD (cholinesterases)^58^. In the present study, the effects of the investigated extracts on selected cholinesterases were assessed by microtiter assays, the results being depicted in Table 2. The aqueous infusion showed better cholinesterase activities, (2.12 ± 0.03 and 1.65 ± 0.06 GALAE per gram extract). The values are thrice higher than those reported for leaves of the known medicinal plant *Lycium barbarum* (Goji) (1.02 ± 0.17 mg GALAE/g dry weight). However, chlorogenic acid, one of the constituent of this plant, has been reported as a potent enzyme inhibitory constituent of Goji leaves^34^ and blueberries^42^. Another Asteraceae species (*Xeranthemum annuum* L.) showed also BCHe inhibitory activity (the chloroform, ethanol and ethyl acetate extracts of the aerial parts showed more than 90% inhibition, superior even than galantamine, which showed 90% inhibition)^59^. Other Asteraceae species, such as wild artichoke (*Cynara cornigera*), which containt sesquiterpene lactones, showed also high-inhibitory activity^60^. *Calendula* extracts (*n*-hexane, dichloromethane, acetone, ethyl acetate, methanol, and water) of the leaf and flowers of *Calendula arvensis* L. and *C. officinalis* L. were also inhibitors of acetylcholinesterase (AChE) and butyrylcholinesterase (BChE)^61^.

## Conclusions

The infusion of the endemic plant *L. rivularis,* which is used in the Chilean traditional system of medicine for gastrointestinal ailments and prevention of cancer, showed antioxidant and cholinesterase inhibitory activities. Several phenolic and other interesting compounds were identified in the infusion of this important Mapuche species for the first time. Among the compounds detected, 4 were sesquiterpenes (peaks 34, 25, 28, and 29), 10 were flavonoids (peaks 10–13, 18, 20, 26, 24, 35, and 37), 9 were oxylipins (peaks 22, 23, 27, 30, 31, 32, and 36), 2 were organic acids (peaks 1 and 2), and 11 were phenolic acids (peaks 3–9, 14, 15, 17, and 33). The results confirm that this plant is a rich source of phenolic compounds that could be responsible for the bioactivity reported and the medicinal use. The infusion and its active components could emerge as natural antioxidants, or serve as raw materials for the isolation of effective AChE inhibitors, thus being a promising potential complementary source against Alzheimer's disease and related diseases.

## Supplementary Material

IENZ_1466880_Supplementary_Material.pdf

## References

[1] Martinez R, Kesternich V, Carrasco H, et al Structure, conformation and biological activity studies on rivularin, a new heliangolide isolated from *Leptocarpha rivularis*. Bol Soc Chilena Quim 1998;43:7–12.

[2] Martinez R, Ayamante IS, Nunezalarcon JA, et al Leptocarpin and 17,18-dihydroleptocarpin, 2 new heliangolides from *Leptocarpha rivularis*. Phytochemistry 1979;18:1527–8.

[3] Bosio C, Tomasoni G, Martínez R, et al Cytotoxic and apoptotic effects of leptocarpin, a plant-derived sesquiterpene lactone, on human cancer cell lines. Chem Biol Interact 2015;242:415–21. 26562779 10.1016/j.cbi.2015.11.006

[4] Uquiche E, Garcés F. Recovery and antioxidant activity of extracts from *Leptocarpha rivularis* by supercritical carbon dioxide extraction. J Supercrit Fluids 2016;110:257–64.

[5] Niemeyer HM. Composition of essential oils from five aromatic species of asteraceae. J Essent Oil Res 2009;21:350–3.

[6] Amorati R, Foti MC, Valgimigli L. Antioxidant activity of essential oils. J Agric Food Chem 2013;61:10835–47. 24156356 10.1021/jf403496k

[7] Spinola V, Llorent-Martinez EJ, Gouveia S, et al *Myrica faya*: a new source of antioxidant phytochemicals. J Agric Food Chem 2014;62:9722–35. 25266067 10.1021/jf503540s

[8] Prameela K, Venkatesh K, Immandi SB, et al Next generation nutraceutical from shrimp waste: the convergence of applications with extraction methods. Food Chem 2017; 237:121–32. 28763972 10.1016/j.foodchem.2017.05.097

[9] Aarli JA, Dua T, Janca A, et al, Neurological disorders. Public health challenges. Geneva: World Health Organization; 2006.

[10] Gotz J, Ittner A, Ittner LM. Tau-targeted treatment strategies in Alzheimer's disease. Br J Pharmacol 2012;165:1246–59. 22044248 10.1111/j.1476-5381.2011.01713.x PMC3372713

[11] Ballard C, Gauthier S, Corbett A, et al Alzheimer's disease. Lancet 2011; 377:1019–31. 21371747 10.1016/S0140-6736(10)61349-9

[12] Yi L, Liu W, Wang Z, et al Characterizing Alzheimer's disease through metabolomics and investigating anti-Alzheimer's disease effects of natural products. Ann New York Acad Sci 2017;1398:130–41. 28632966 10.1111/nyas.13385

[13] Dey A, Bhattacharya R, Mukherjee A, et al Natural products against Alzheimer's disease: pharmaco-therapeutics and biotechnological interventions. Biotechnol Adv 2017;35:178–216. 28043897 10.1016/j.biotechadv.2016.12.005

[14] Bassil N, Grossberg GT. Novel regimens and delivery systems in the pharmacological treatment of Alzheimer's Disease. CNS Drugs 2009;23:293–307. 19374459 10.2165/00023210-200923040-00003

[15] Custódio L, Patarra J, Alberício F, et al Extracts from *Quercus* sp. acorns exhibit *in vitro* neuroprotective features through inhibition of cholinesterase and protection of the human dopaminergic cell line SH-SY5Y from hydrogen peroxide-induced cytotoxicity. Ind Crops Prod 2013;45:114–20.

[16] Trung Kien N, Kyung Hoan L, Jaehyuk C, et al Evaluation of antioxidant, anti-cholinesterase, and anti-inflammatory effects of culinary mushroom *Pleurotus pulmonarius*. Mycobiology 2016;44:291–301. 28154487 10.5941/MYCO.2016.44.4.291 PMC5287162

[17] Melucci D, Locatelli M, Locatelli C, et al A comparative assessment of biological effects and chemical profile of Italian *Asphodeline lutea* extracts. Molecules 2018;23:461. 10.3390/molecules23020461 PMC6017467 29463056

[18] Le Pogam P, Schinkovitz A, Legouin B, et al Matrix-Free UV-laser desorption ionization mass spectrometry as a versatile approach for accelerating dereplication studies on Lichens. Anal Chem 2015;87:10421–8. 26378462 10.1021/acs.analchem.5b02531

[19] Musharraf SG, Kanwal N, Thadhani VM, et al Rapid identification of lichen compounds based on the structure-fragmentation relationship using ESI-MS/MS analysis. Anal Meth 2015;7:6066–76.

[20] Cornejo A, Salgado F, Caballero J, et al Secondary metabolites in *Ramalina terebrata* detected by UHPLC/ESI/MS/MS and identification of parietin as Tau protein inhibitor. Int J Mol Sci 2016;17:1703. 10.3390/ijms17081303 PMC5000700 27548142

[21] Castro ON, Benites J, Rodilla J, et al Metabolomic analysis of the Lichen *Everniopsis trulla* using ultra high performance liquid chromatography-quadrupole-Orbitrap Mass Spectro-metry (UHPLC-Q-OT-MS). Chromatographia 2017;80:967–73.

[22] Simirgiotis MJ, Quispe C, Bórquez J, et al Fast high resolution Orbitrap MS fingerprinting of the resin of *Heliotropium taltalense* Phil. from the Atacama Desert. Ind Crops Prod 2016;85:159–66.

[23] Sepulveda B, Quispe C, Simirgiotis M, et al Gastroprotective effects of new diterpenoid derivatives from *Azorella cuatrecasasii* Mathias & Constance obtained using a β-cyclodextrin complex with microbial and chemical transformations. Bioorg Med Chem Lett 2016;26:3220–2. 27262597 10.1016/j.bmcl.2016.05.081

[24] Simirgiotis MJ, Bórquez J, Neves-Vieira M, et al Fast isolation of cytotoxic compounds from the native Chilean species *Gypothamnium pinifolium* Phil. collected in the Atacama Desert, northern Chile. Ind Crops Prod 2015;76:69–76.

[25] Simirgiotis MJ, Quispe C, Areche C, et al Phenolic compounds in Chilean Mistletoe (Quintral, Tristerix tetrandus) analyzed by UHPLC-Q/Orbitrap/MS/MS and its antioxidant properties. Molecules 2016;21:245. 26907248 10.3390/molecules21030245 PMC6274319

[26] Simirgiotis MJ, Quispe C, Bórquez J, et al Fast detection of phenolic compounds in extracts of Easter pears (*Pyrus communis*) from the Atacama Desert by ultrahigh-performance liquid chromatography and mass spectrometry (UHPLC-Q/Orbitrap/MS/MS). Molecules 2016;21:92–105. 26784158 10.3390/molecules21010092 PMC6273977

[27] Simirgiotis MJ, Ramirez JE, Hirschmann GS, et al Bioactive coumarins and HPLC-PDA-ESI-ToF-MS metabolic profiling of edible queule fruits (*Gomortega keule*), an endangered endemic Chilean species. Food Res Int 2013;54:532–43.

[28] Brand-Williams W, Cuvelier ME, Berset C. Use of a free radical method to evaluate antioxidant activity. LWT – Food Sci Technol 1995;28:25–30.

[29] Kim DO, Lee KW, Lee HJ, et al Vitamin C equivalent antioxidant capacity (VCEAC) of phenolic phytochemicals. J Agric Food Chem 2002;50:3713–7. 12059148 10.1021/jf020071c

[30] Kuskoski EM, Asuero AG, García-Parilla MC, et al Actividad antioxidante de pigmentos antociánicos. Food Sci Technol 2004;24:691–3.

[31] Benzie IFF, Strain JJ. The ferric reducing ability of plasma (FRAP) as a measure of “antioxidant power”: the FRAP assay. Anal Biochem 1996;239:70–6. 8660627 10.1006/abio.1996.0292

[32] Simirgiotis MJ, Borquez J, Schmeda-Hirschmann G Antioxidant capacity, polyphenolic content and tandem HPLC-DAD-ESI/MS profiling of phenolic compounds from the South American berries *Luma apiculata* and *L*. *chequén*. Food Chem 2013;139:289–99. 23561108 10.1016/j.foodchem.2013.01.089

[33] Aktumsek A, Zengin G, Guler GO, et al Antioxidant potentials and anticholinesterase activities of methanolic and aqueous extracts of three endemic Centaurea L. species. Food Chem Toxicol 2013;55:290–6. 23357566 10.1016/j.fct.2013.01.018

[34] Llorent-Martinez EJ, Zengin G, Fernandez-de Cordova ML, et al Traditionally used *Lathyrus* species: phytochemical composition, antioxidant activity, enzyme inhibitory properties, cytotoxic effects, and *in silico* studies of *L. czeczottianus* and *L. nissolia*. Front Pharmacol 2017;8:83. 28289386 10.3389/fphar.2017.00083 PMC5326780

[35] Wu H, Huang T, Cao F, et al Co-production of HMF and gluconic acid from sucrose by chemo-enzymatic method. Chem Eng J 2017;327:228–34.

[36] Brito A, Ramirez JE, Areche C, et al HPLC-UV-MS profiles of phenolic compounds and antioxidant activity of fruits from three citrus species consumed in Northern Chile. Molecules 2014;19:17400–21. 25356563 10.3390/molecules191117400 PMC6271594

[37] Abu Irmaileh BE, Al-Aboudi AMF, Abu Zarga MH, et al Selective phytotoxic activity of 2,3,11 beta,13-tetrahydroaromaticin and ilicic acid isolated from *Inula graveolens*. Nat Prod Res 2015;29:893–8. 25190268 10.1080/14786419.2014.955489

[38] Nam MH, Son WR, Yang SY, et al Chebulic acid inhibits advanced glycation end products-mediated vascular dysfunction by suppressing ROS via the ERK/Nrf2 pathway. J Funct Foods 2017;36:150–61.

[39] Sonmezdag AS, Kelebek H, Selli S. Pistachio oil (*Pistacia vera* L. cv. Uzun): characterization of key odorants in a representative aromatic extract by GC-MS-olfactometry and phenolic profile by LC-ESI-MS/MS. Food Chem 2018;240:24–31. 28946268 10.1016/j.foodchem.2017.07.086

[40] Kent PW, Brunet PCJ. The occurrence of protocatechuic acid and its 4-O-β-d-glucoside in Blatta and Periplaneta. Tetrahedron 1959;7:252–6.

[41] Silva RV, Costa SCC, Branco CRC, et al *In vitro* photoprotective activity of the *Spondias purpurea* L. peel crude extract and its incorporation in a pharmaceutical formulation. Ind Crops Prod 2016;83:509–14.

[42] Mollica A, Locatelli M, Macedonio G, et al Microwave-assisted extraction, HPLC analysis, and inhibitory effects on carbonic anhydrase I, II, VA, and VII isoforms of 14 blueberry Italian cultivars. J Enz Inhib Med Chem 2016;31:1–6. 10.1080/14756366.2016.1214951 27541737

[43] Willems JL, Khamis MM, Mohammed Saeid W, et al Analysis of a series of chlorogenic acid isomers using differential ion mobility and tandem mass spectrometry. Anal Chim Acta 2016;933:164–74. 27497009 10.1016/j.aca.2016.05.041

[44] Clifford MN, Knight S, Kuhnert N. Discriminating between the six isomers of dicaffeoylquinic acid by LC-MS(n). J Agric Food Chem 2005;53:3821–32. 15884803 10.1021/jf050046h

[45] Han J, Wang D, Ye L, et al Rosmarinic acid protects against inflammation and cardiomyocyte apoptosis during myocardial ischemia/reperfusion injury by activating peroxisome proliferator-activated receptor gamma. Front Pharmacol 2017;8. 10.3389/fphar.2017.00456 PMC5504166 28744220

[46] Simirgiotis MJ. Antioxidant capacity and HPLC-DAD-MS profiling of Chilean Peumo (*Cryptocarya alba*) fruits and comparison with German Peumo (*Crataegus monogyna*) from Southern Chile. Molecules 2013;18:2061–80. 23385342 10.3390/molecules18022061 PMC6270219

[47] Simirgiotis MJ, Benites J, Areche C, et al Antioxidant capacities and analysis of phenolic compounds in three endemic *Nolana* species by HPLC-PDA-ESI-MS. Molecules 2015;20:11490–507. 26111178 10.3390/molecules200611490 PMC6272610

[48] Eshbakova K, Yili A, Aisa H. Phenolic Constituents of *Pulicaria gnaphaloides*. Chem Nat Comp 2014;50:737–8.

[49] Liu D-Y, Shi X-F, Wang D-d, et al Two new myricetin glycosides from pine needles of *Cedrus deodara*. Chem Nat Comp 2011;47:704–7.

[50] Veitch NC, Grayer RJ. Flavonoids and their glycosides, including anthocyanins. Nat Prod Rep 2008;25:555–611. 18497898 10.1039/b718040n

[51] Jie LIU, REN H, Bin LIU, et al Diosmetin inhibits cell proliferation and induces apoptosis by regulating autophagy via the mammalian target of rapamycin pathway in hepatocellular carcinoma HepG2 cells. Oncol Lett 2016;12:4385–92. 28101201 10.3892/ol.2016.5301 PMC5228182

[52] Jiménez-Sánchez C, Lozano-Sánchez J, Rodríguez-Pérez C, et al Comprehensive, untargeted, and qualitative RP-HPLC-ESI-QTOF/MS2 metabolite profiling of green asparagus (*Asparagus officinalis*). J Food Comp Anal 2016;46:78–87.

[53] Hu WJ, Pan XL, Abbas HMK, et al Metabolites contributing to *Rhizoctonia solani* AG-1-IA maturation and sclerotial differentiation revealed by UPLC-QTOF-MS metabolomics. PloS One 2017;12:e0177464. 28489938 10.1371/journal.pone.0177464 PMC5425210

[54] Zengin G, Uysal A, Aktumsek A, et al *Euphorbia denticulata* Lam.: a promising source of phyto-pharmaceuticals for the development of novel functional formulations. Biomed Pharmacother 2017;87:27–36. 28040595 10.1016/j.biopha.2016.12.063

[55] Martin-Arjol I, Bassas M, Bermudo E, et al Identification of oxylipins with antifungal activity by LC-MS/MS from the supernatant of *Pseudomonas* 42A2. Chem Phys Lip 2010;163:341–6. 10.1016/j.chemphyslip.2010.02.003 20188718

[56] Mocan A, Moldovan C, Zengin G, et al UHPLC-QTOF-MS analysis of bioactive constituents from two Romanian Goji (*Lycium barbarum* L.) berries cultivars and their antioxidant, enzyme inhibitory, and real-time cytotoxicological evaluation. Food Chem Toxicol 2018;115:414–24. 29448090 10.1016/j.fct.2018.01.054

[57] Mocan A, Zengin G, Simirgiotis M, et al Functional constituents of wild and cultivated Goji (*L. barbarum* L.) leaves: phytochemical characterization, biological profile, and computational studies. J Enz Inhibit Med Chem 2017;32:153–68., 10.1080/14756366.2016.1243535 PMC6009880 28095717

[58] Seo WD, Kim JY, Ryu HW, et al Identification and characterisation of coumarins from the roots of *Angelica dahurica* and their inhibitory effects against cholinesterase. J Funct Foods 2013;5:1421–31.

[59] Orhan IE, Gulyurdu F, Kupeli Akkol E, et al Anticholinesterase, antioxidant, analgesic and anti-inflammatory activity assessment of *Xeranthemum annuum* L. and isolation of two cyanogenic compounds. Pharm Biol 2016;54:2643–51. 27465673 10.1080/13880209.2016.1177092

[60] Hegazy M-EF, Ibrahim AY, Mohamed TA, et al Sesquiterpene lactones from *Cynara cornigera*: acetyl cholinesterase inhibition and *in silico* ligand docking. Planta Med 2016;82:138–45. 26441064 10.1055/s-0035-1558088

[61] Ercetin T, Senol FS, Erdogan Orhan I, et al Comparative assessment of antioxidant and cholinesterase inhibitory properties of the marigold extracts from Calendula arvensis L. and *Calendula officinalis* L. Ind Crops Prod 2012;36:203–8.

[62] Szwajgier D. Anticholinesterase activity of selected phenolic acids and flavonoids – interaction testing in model solutions. Ann Agric Environ Med 2015;22:690–4. 26706979 10.5604/12321966.1185777

