# Supplementary material for: UHPLC-ESI-ORBITRAP-MS analysis of the native Mapuche medicinal plant palo negro (Leptocarpha rivularis DC. – Asteraceae) and evaluation of its antioxidant and cholinesterase inhibitory properties
Source: J Enzyme Inhib Med Chem. 2018 May 7;33(1):936–44. doi: 10.1080/14756366.2018.1466880 (PMC6009940; doi:10.1080/14756366.2018.1466880)
Supplement: IENZ_1466880_Supplementary_Material.pdf [file IENZ_A_1466880_SM7650.pdf]

UHPLC-ESI-ORBITRAP-MS-MS analysis of the Native Mapuche Medicinal Plant Palo Negro (*Leptocarpha rivularis* DC. – Asteraceae) and evaluation of its antioxidant and cholinesterase inhibitory properties

Andrea Jiménez-Gonzalez[a], Cristina Quispe[b], Jorge Bórquez [c], Beatriz Sepúlveda [d], Felipe Riveros[c], Carlos Areche [e], Edgar Nagles [a], Olimpo García-Beltrán [a]*, and Mario J. Simirgiotis [f,g]*

[a] *Facultad de Ciencias Naturales y Matematicas, Universidad de Ibague, Carrera 22 calle 67, Ibagué 730001, Colombia*

[b] *Instituto de Etnofarmacología, Universidad Arturo Prat, Facultad de Ciencias de la Salud, Casilla 121, Iquique, 1110939, Chile.*

[c] *Laboratorio de Productos Naturales, Departamento de Química, Facultad de Ciencias Básicas, Universidad de Antofagasta, 1240000, Chile*

[d] *Departamento de Ciencias Químicas, Universidad Andres Bello, Campus Viña del Mar, Quillota 980, Viña del Mar 2520000, Chile.*

[e] *Departamento de Química, Facultad de Ciencias, Universidad de Chile, Casilla 653, Santiago 7800024, Chile*

[f] *Instituto de Farmacia, Facultad de Ciencias, Universidad Austral de Chile, Campus Isla Teja 5090000, Valdivia.*

[g] *Center for Interdisciplinary Studies on the Nervous System, Universidad Austral de Chile, Campus Isla Teja 5090000, Valdivia.*

Corresponding authors: Olimpo García-Beltrán: Facultad de Ciencias Naturales y Matematicas, Universidad de Ibague, Carrera 22 calle 67, Ibagué 730001; Mario Simirgiotis: Instituto de Farmacia, Facultad de Ciencias, Universidad Austral de Chile, Campus Isla Teja 5090000, Valdivia; e mail: mario.simirgiotis@uach.cl.

Fig. S1 (a-l): Full Orbitrap HR-MS spectra and proposed structures of compounds 11, 13, 15, 16, 24, 26, 28 31- 35.

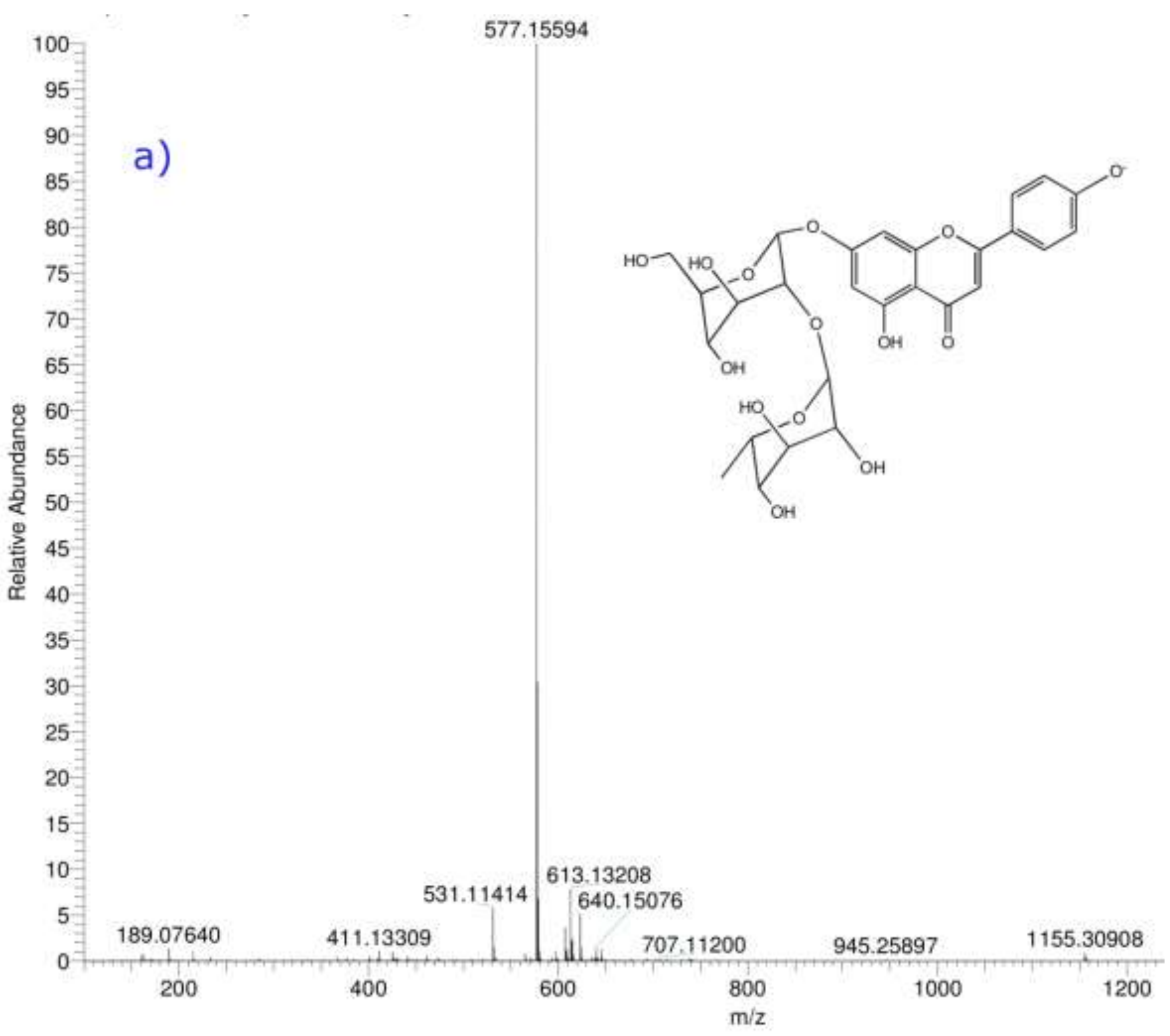

577.15594
a)
Relative Abundance
531.11414
613.13208
640.15076
189.07640
411.13309
707.11200
945.25897
1155.30908
m/z

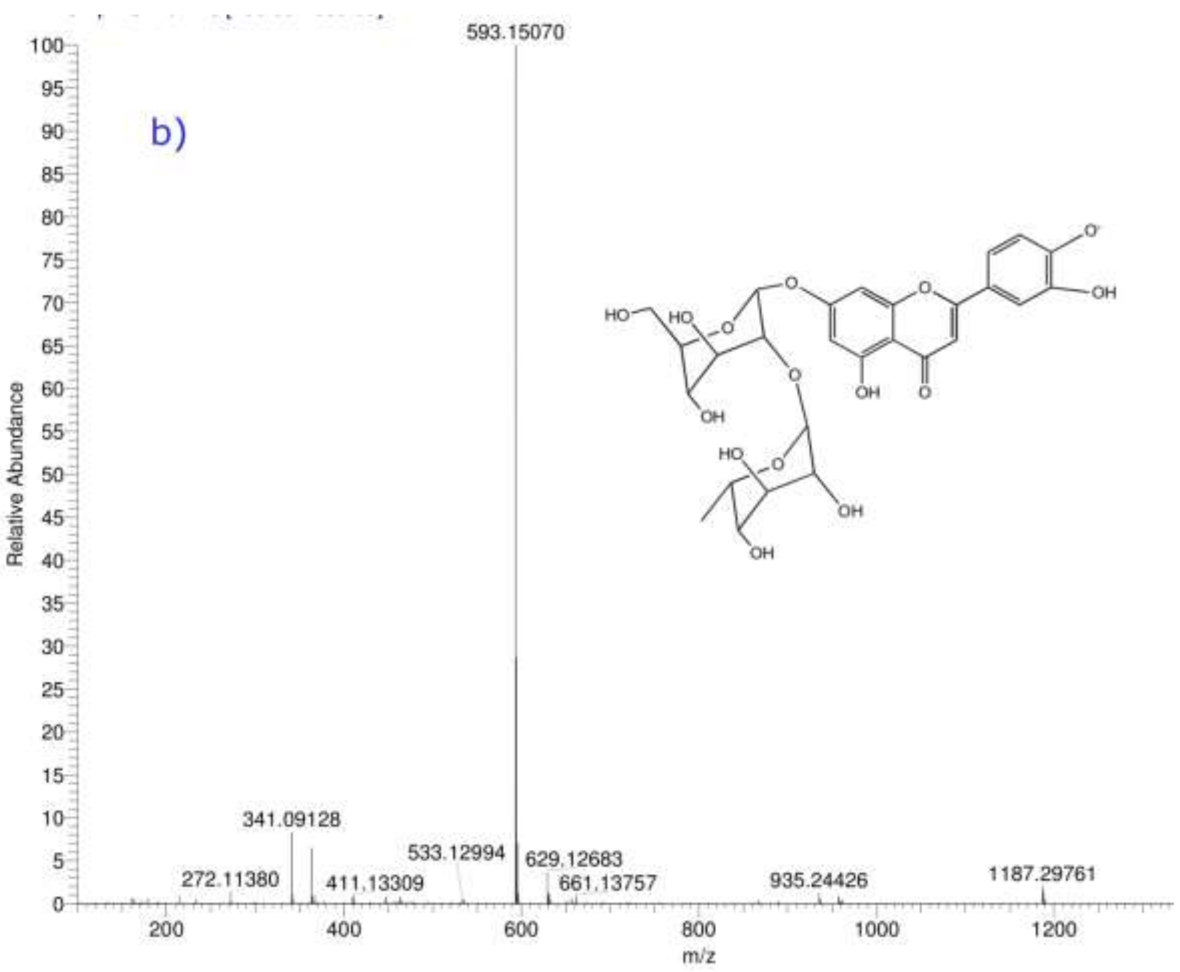

593.15070
b)
100
95
90
85
80
75
70
65
60
55
50
45
40
35
30
25
20
15
10
5
0
Relative Abundance
341.09128
272.11380
411.13309
533.12994
629.12683
661.13757
935.24426
1187.29761
200
400
600
800
1000
1200
m/z
HO
HO
O
OH
OH
O
O
O
OH
O
OH
O⁻
HO
O
OH
OH

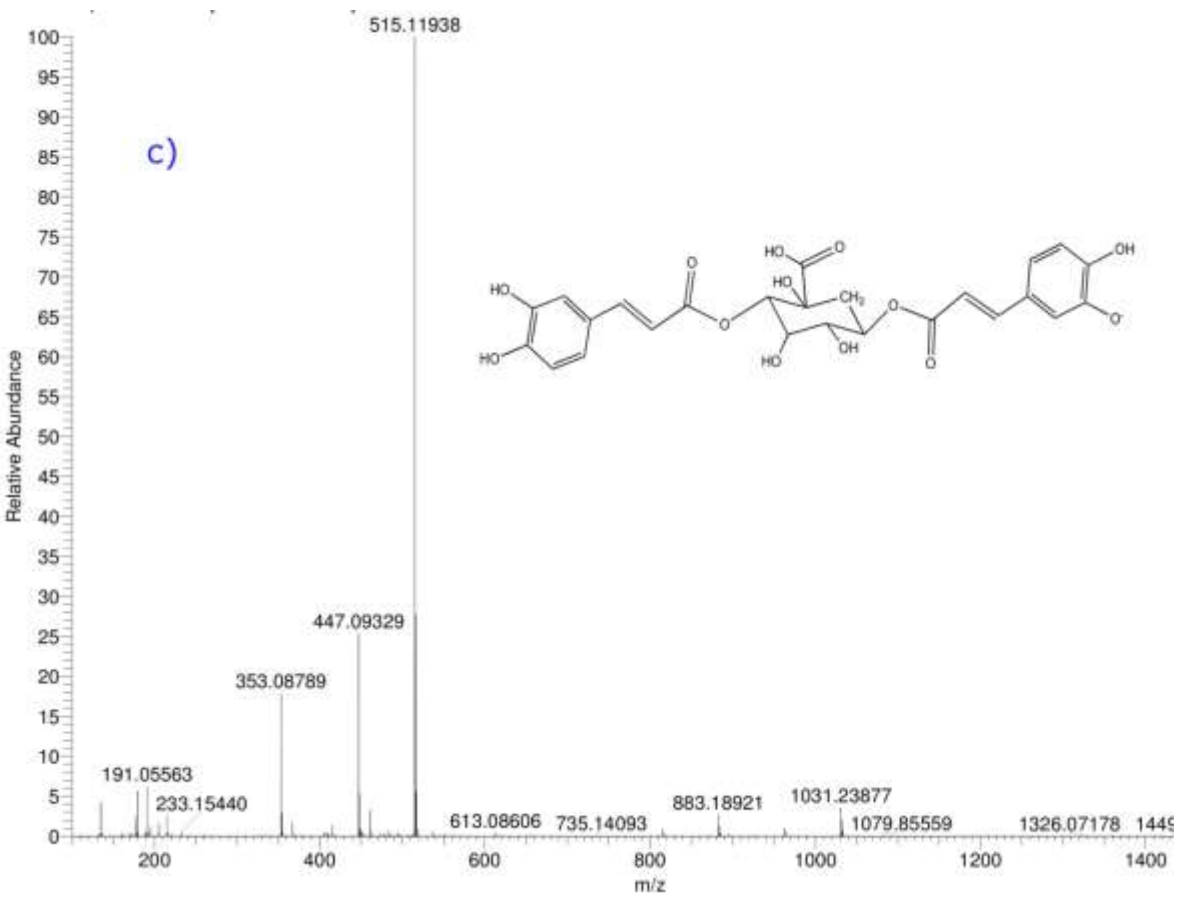
c)
515.11938
447.09329
353.08789
191.05563
233.15440
613.08606
735.14093
883.18921
1031.23877
1079.85559
1326.07178
Relative Abundance
m/z
HO
HO
OH
O
O
O
HO
HO
CH3
OH
HO
O
O
OH
O-

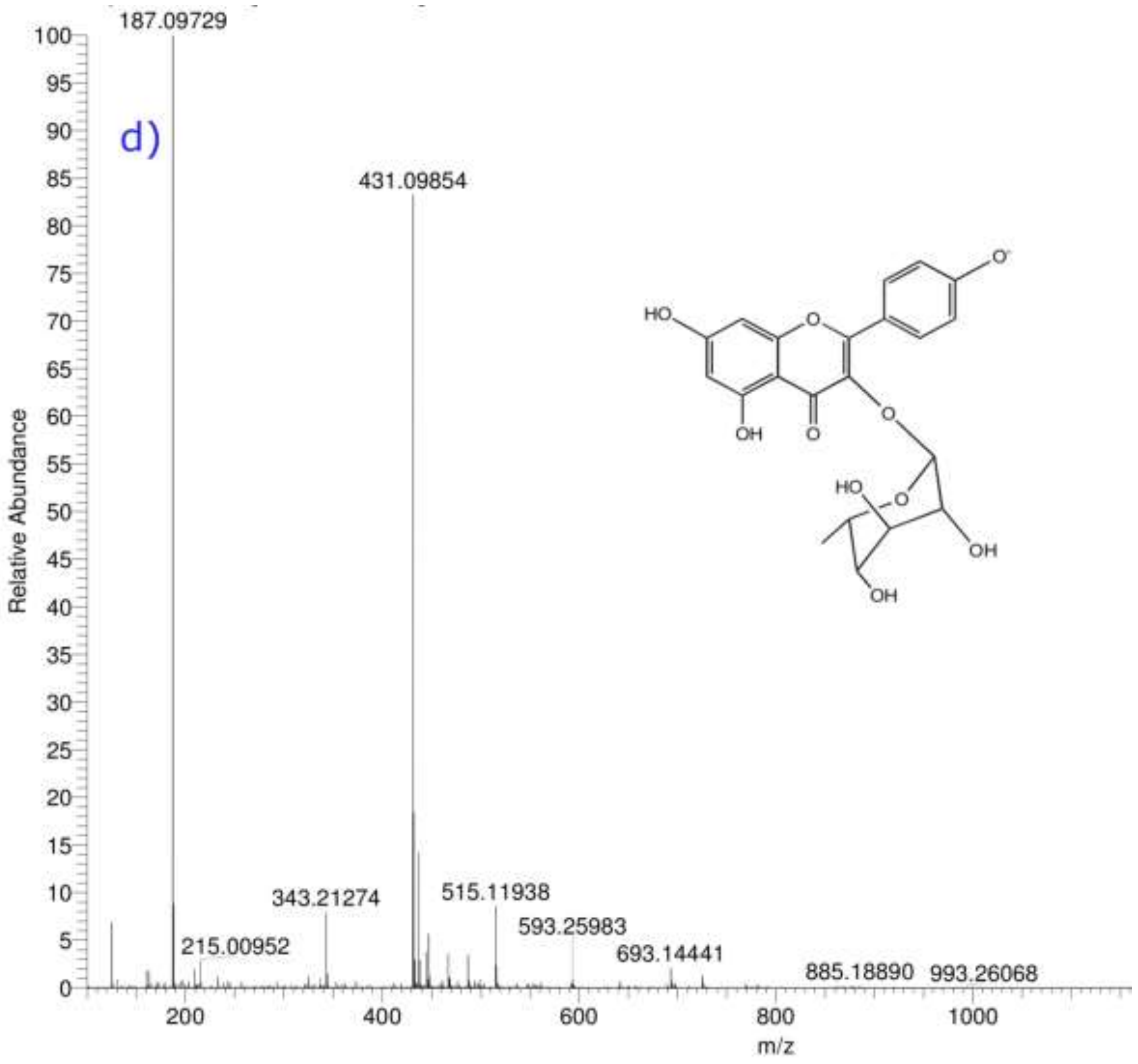
187.09729
d)
431.09854
343.21274
215.00952
515.11938
593.25983
693.14441
885.18890
993.26068
Relative Abundance
m/z
HO
O
O⁻
OH
O
O
HO
O
OH
OH

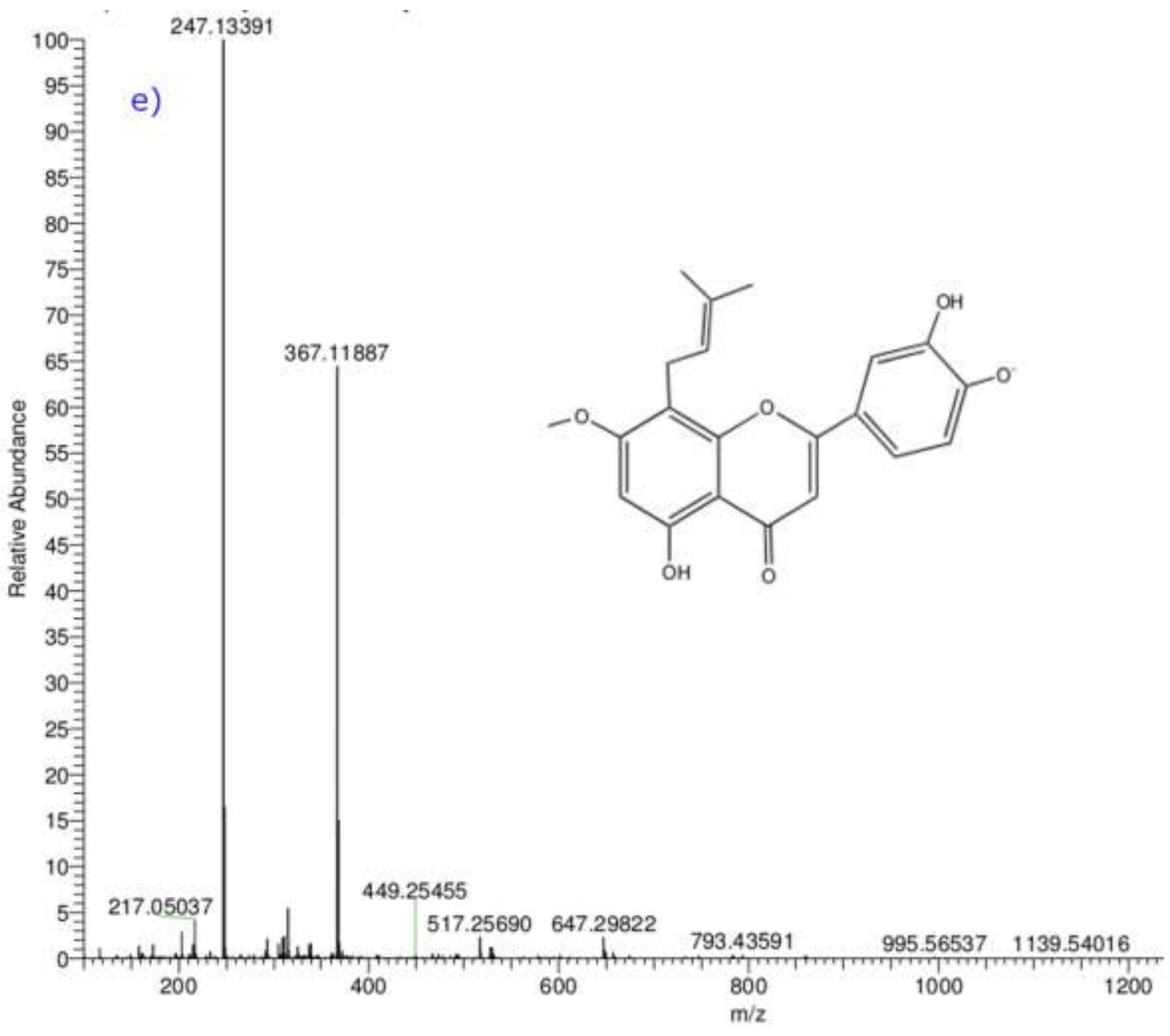
e)
247.13391
367.11887
217.05037
449.25455
517.25690
647.29822
793.43591
995.56537
1139.54016
Relative Abundance
m/z
OH
O
OH
O

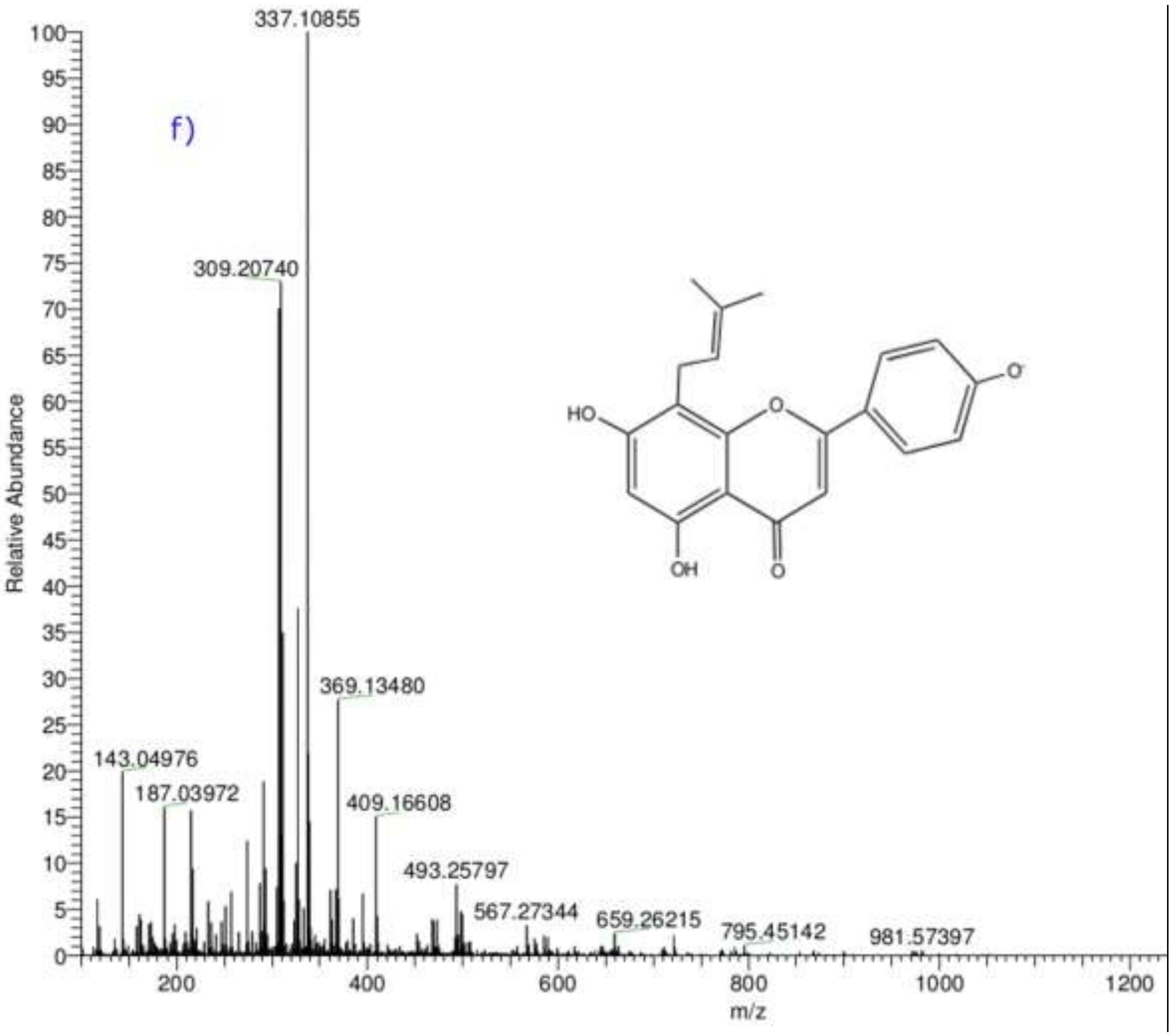
337.10855
f)
309.20740
369.13480
143.04976
187.03972
409.16608
493.25797
567.27344
659.26215
795.45142
981.57397
Relative Abundance
m/z
HO
OH
O
O
O⁻

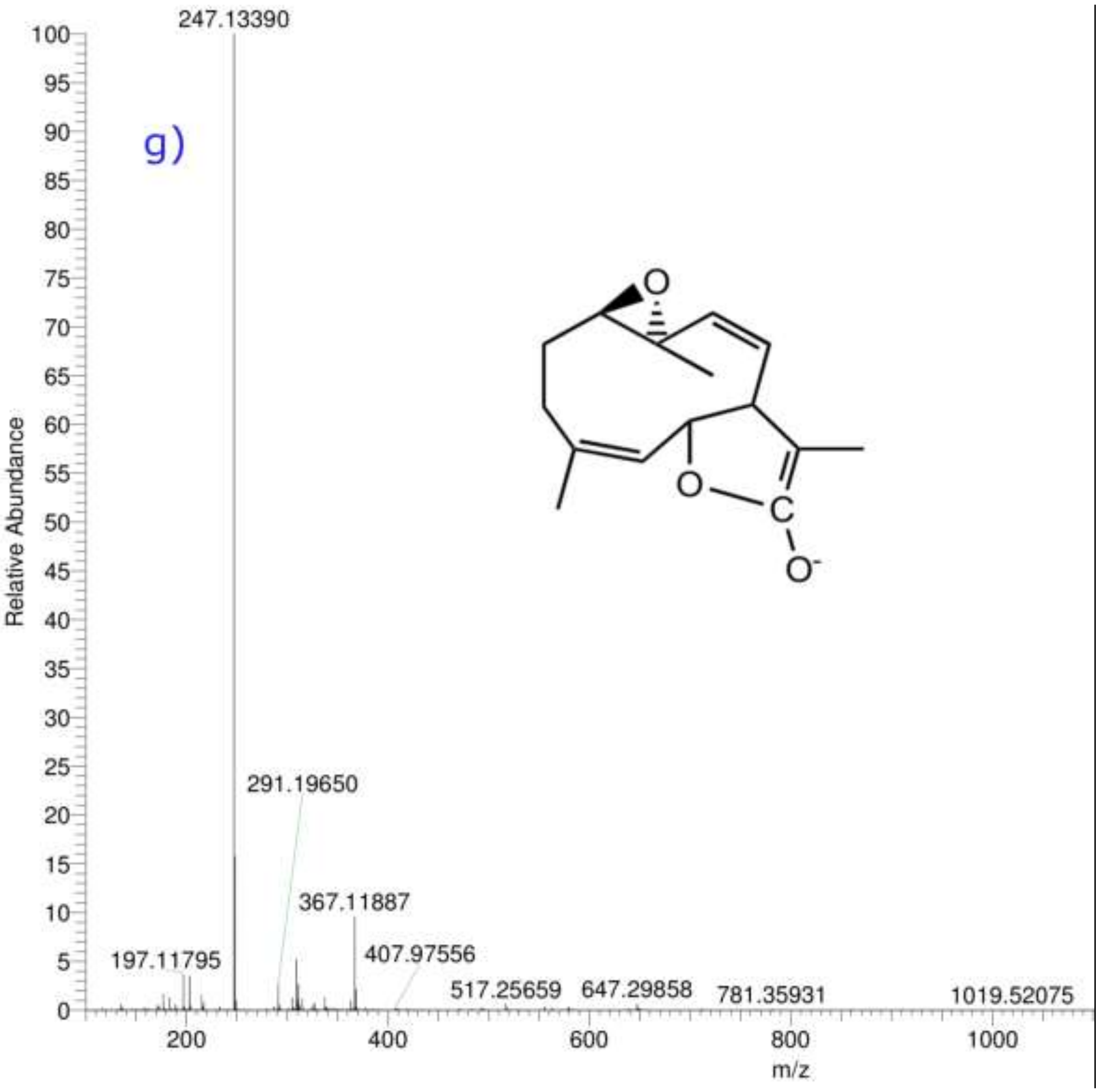
g)
247.13390
291.19650
367.11887
407.97556
197.11795
517.25659
647.29858
781.35931
1019.52075
Relative Abundance
m/z
O
O
C
O-

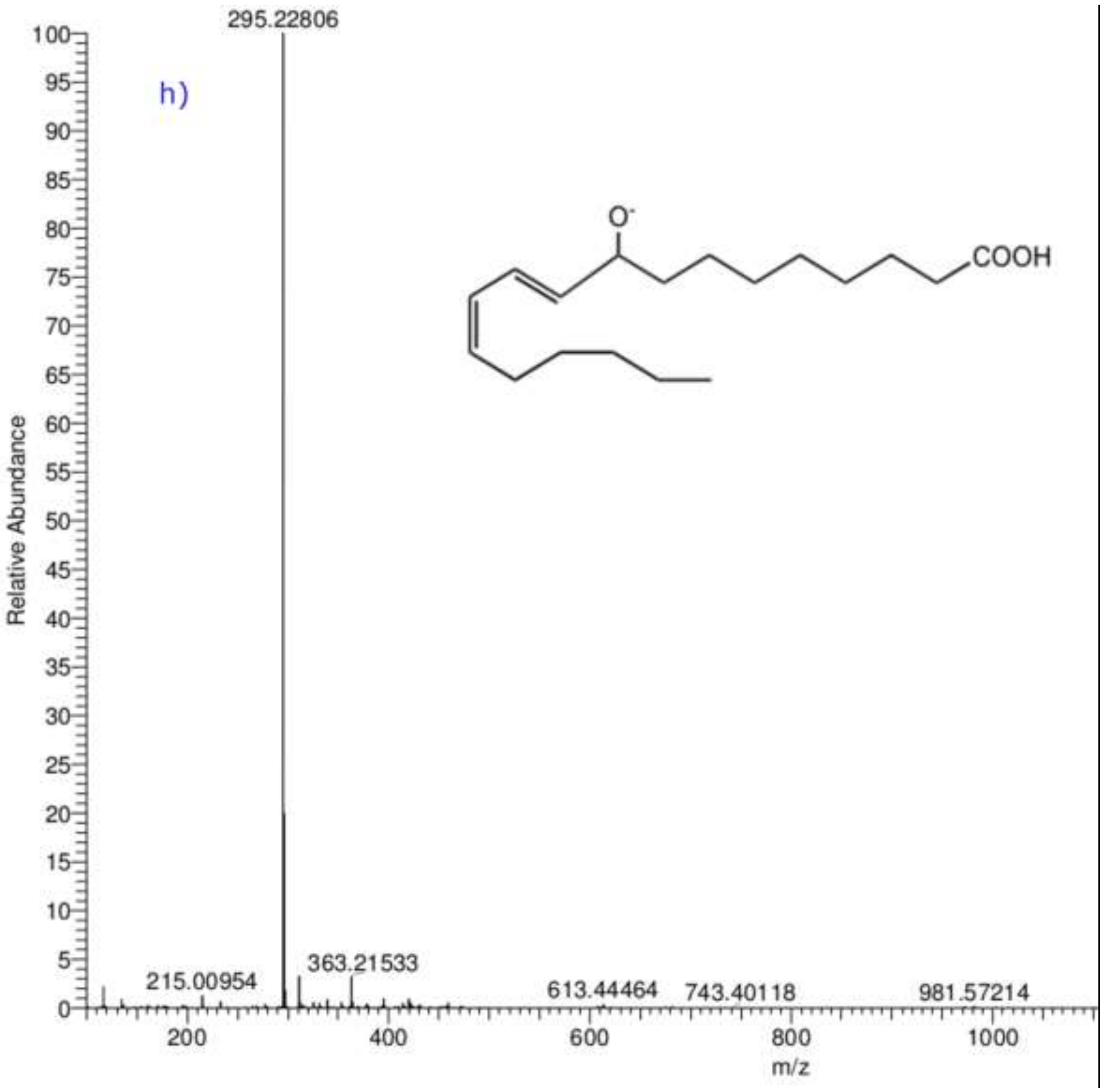

295.22806
h)
O⁻
COOH
215.00954
363.21533
613.44464
743.40118
981.57214
Relative Abundance
m/z

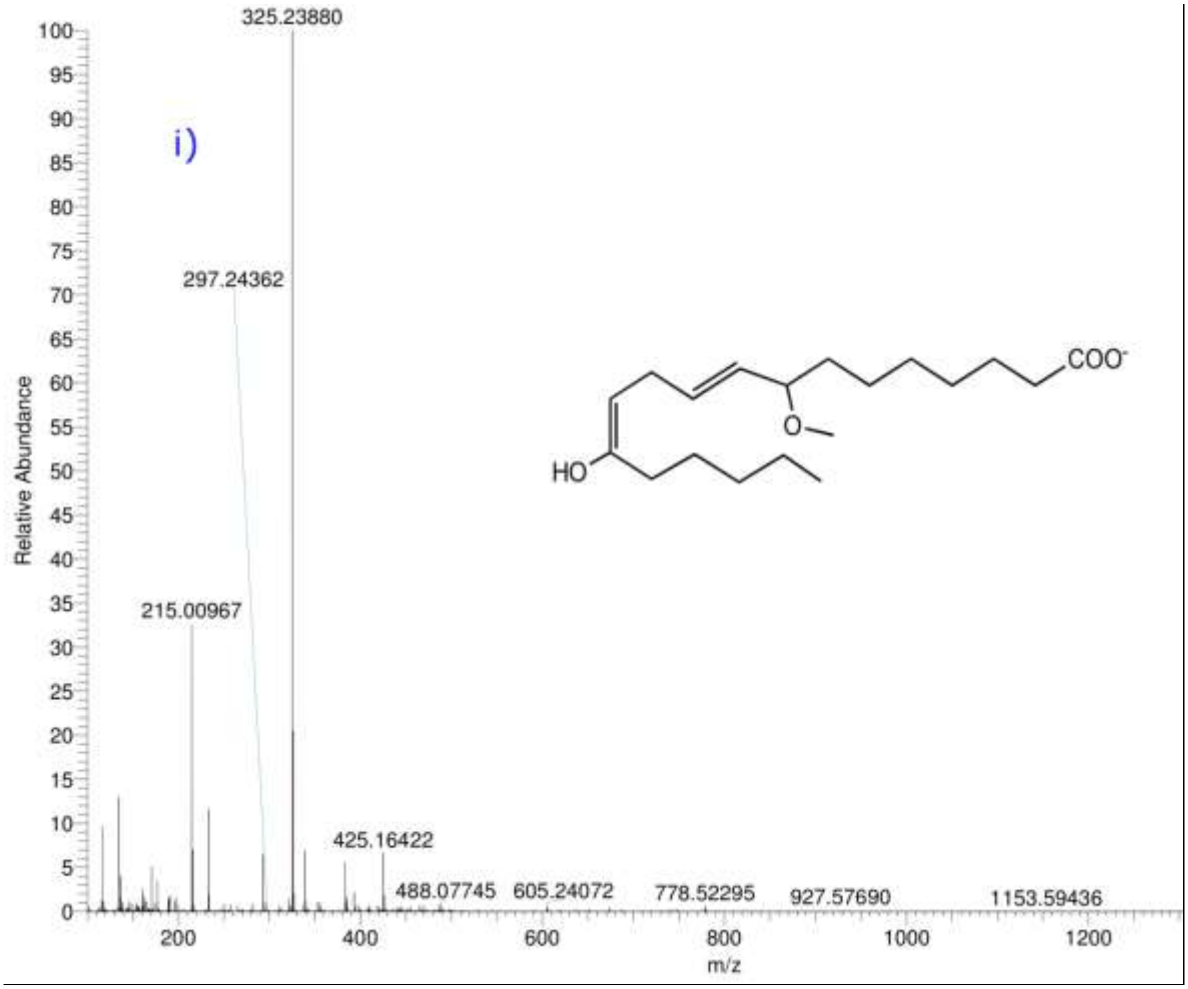

i)
325.23880
297.24362
215.00967
425.16422
488.07745
605.24072
778.52295
927.57690
1153.59436
Relative Abundance
m/z
COO-
HO
O

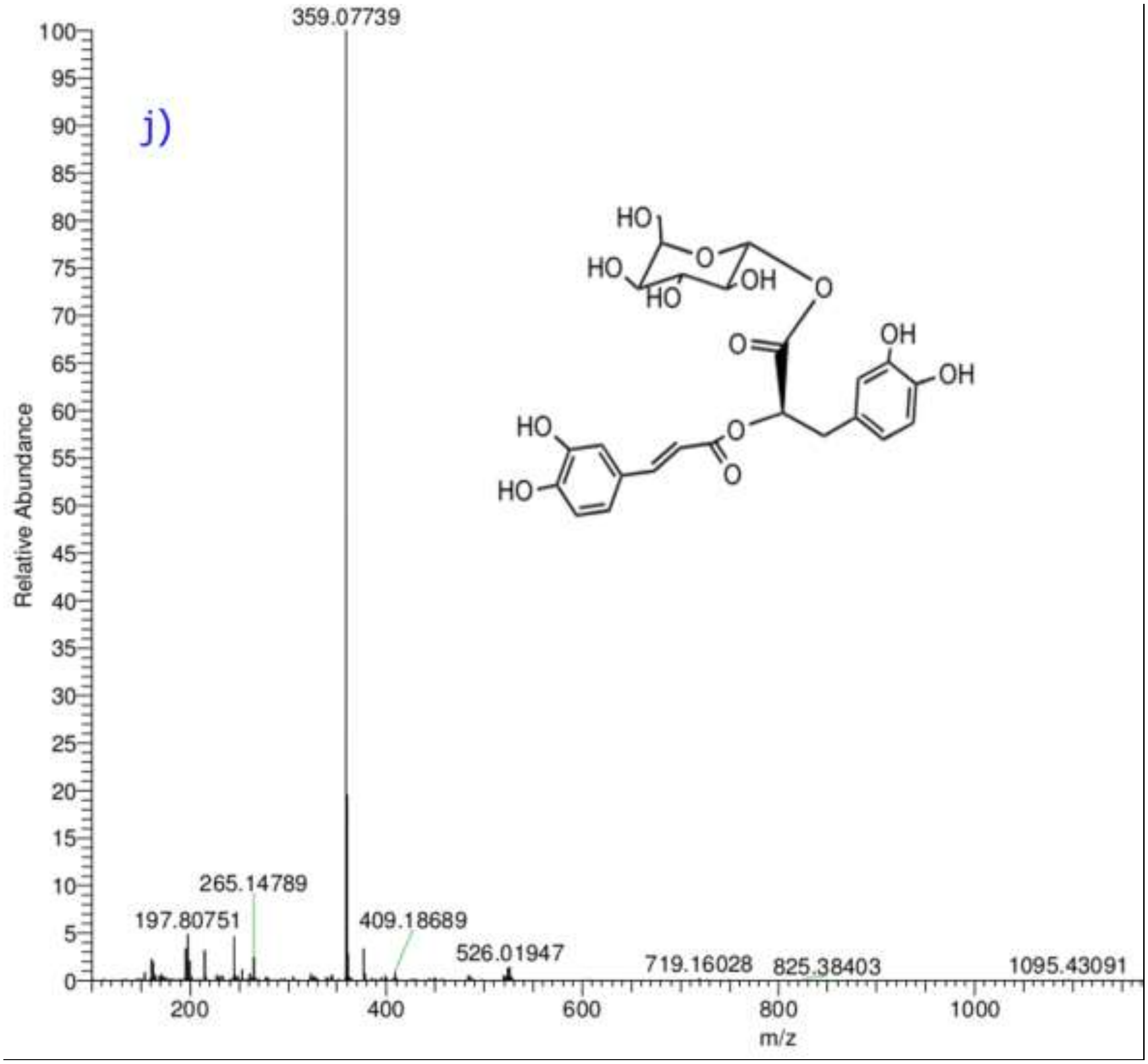
359.07739
j)
Relative Abundance
265.14789
197.80751
409.18689
526.01947
719.16028
825.38403
1095.43091
m/z
HO
O
HO
OH
HO
O
O
OH
OH
HO
O
HO
O

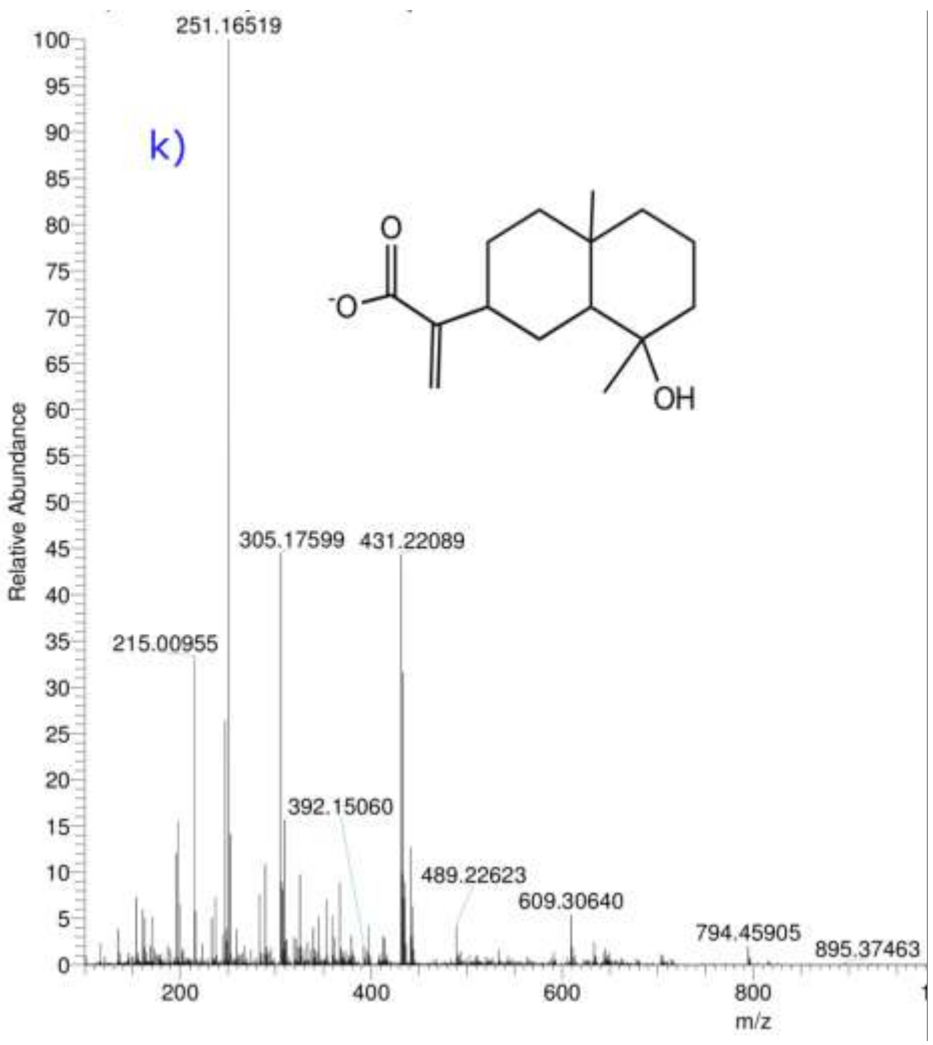
k)
251.16519
305.17599
431.22089
215.00955
392.15060
489.22623
609.30640
794.45905
895.37463
Relative Abundance
m/z
O
-O
OH

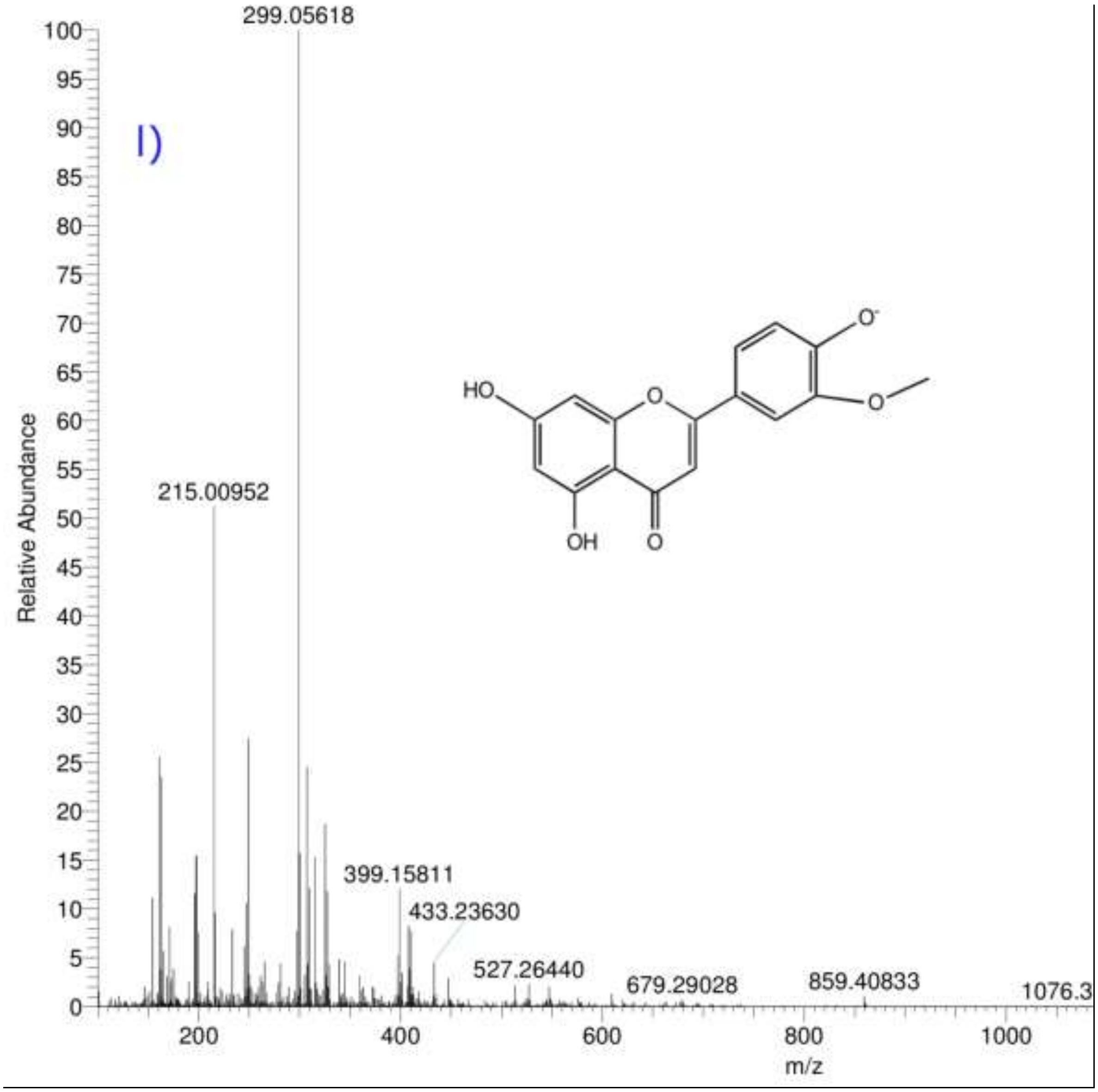
I)
299.05618
215.00952
399.15811
433.23630
527.26440
679.29028
859.40833
1076.3
Relative Abundance
m/z
HO
O
O⁻
OH
O
O
